# Is there a link between fascia and cancer? From potential mechanisms to future treatment options

**DOI:** 10.3389/fphys.2026.1741526

**Published:** 2026-05-08

**Authors:** Stephanie Otto, Werner Klingler

**Affiliations:** 1 Comprehensive Cancer Center Ulm (CCCU), Ulm University, Ulm, Germany; 2 Department of Gynaecology and Obstetrics, Ulm University, Ulm, Germany; 3 Department of Internal Medicine I, Ulm University, Ulm, Germany; 4 Department of Internal Medicine III, Ulm University, Ulm, Germany; 5 Fascia Research Society (FRS), Burnsville, MN, United States; 6 Fascia Research Group, Department of Neurosurgery, Ulm University, Ulm, Germany; 7 Technical University of Munich (TUM) School of Medicine and Health, Professorship for Conservative and Rehabilitative Orthopaedics, Munich, Germany; 8 Faculty of Health, Queensland University of Technology, Brisbane, QLD, Australia; 9 Department of Anaesthesiology, Stiftung Rehabilitation Heidelberg (SRH) Hospital Sigmaringen, Sigmaringen, Germany

**Keywords:** CAF, cancer, extracellular matrix (ECM), epithelial to mesenchymal transformation (EMT), fascia, fasciacytes, fibrosis, tumor microenvironment (TME)

## Abstract

Fascia, the connective tissue network enveloping muscles, organs, nerves, and vasculature, plays an important role in maintaining structural integrity, biomechanical function, and integrative physiological processes. Traditionally considered a passive support structure, fascia is increasingly discussed as an active component in cancer biology, particularly within the tumor microenvironment (TME), although many mechanistic pathways remain incompletely characterized. Experimental and clinical studies suggest that tumor-associated remodeling of the extracellular matrix (ECM) within fascial tissues can increase tissue stiffness, fibrosis, and desmoplasia, changes that may facilitate tumor progression, invasion, metastasis, and immune escape, and could contribute to resistance against conventional therapies in at least some tumor entities. Cancer-associated fibroblasts (CAFs) appear to be central mediators of these processes by producing collagen, enzymatically modifying ECM structure, and influencing mechanotransduction pathways involving transcriptional regulators such as YAP/TAZ and integrins, thereby promoting pro-malignant cellular phenotypes in model systems. Fascia also interfaces closely with immune and nervous systems, potentially influencing immune cell trafficking, neuroinflammatory signaling, and systemic stress responses via the hypothalamic pituitary adrenal axis and vagal pathways, but these interactions are only partly understood in the context of cancer. Emerging preclinical and early clinical data indicate that physical exercise and movement-based interventions, including controlled stretching and other mechanical therapies, may modulate fascial stiffness and low-grade inflammation, with possible effects on tumor biology and patient-reported outcomes that appear at least partly distinct from those of aerobic exercise, yet this requires confirmation in larger, well-controlled trials. Clinically, patterns of fascial involvement and ECM remodeling correlate with aggressive behavior and poorer outcome in several cancers, suggesting potential prognostic value, although standardized assessment and validation across entities are still lacking. Targeting fascia- and ECM-related components, such as collagen cross-linking enzymes (for example lysyl oxidase), is being explored as a translational strategy to enhance drug delivery and immunotherapy responses, but these approaches remain largely experimental. Priority areas for future research include the development of imaging and molecular tools for more precise fascia assessment, mechanobiological interventions tailored to individual patients, and rigorous clinical trials evaluating fascia-modulating therapies as adjuncts in integrative oncology. In summary, current evidence supports a view of fascia as a dynamic, multifunctional tissue that is likely implicated in cancer progression and therapy response while highlighting substantial knowledge gaps and the need for cautious, hypothesis-driven translation into clinical practice.

## Graphical Abstract

**Figure:**
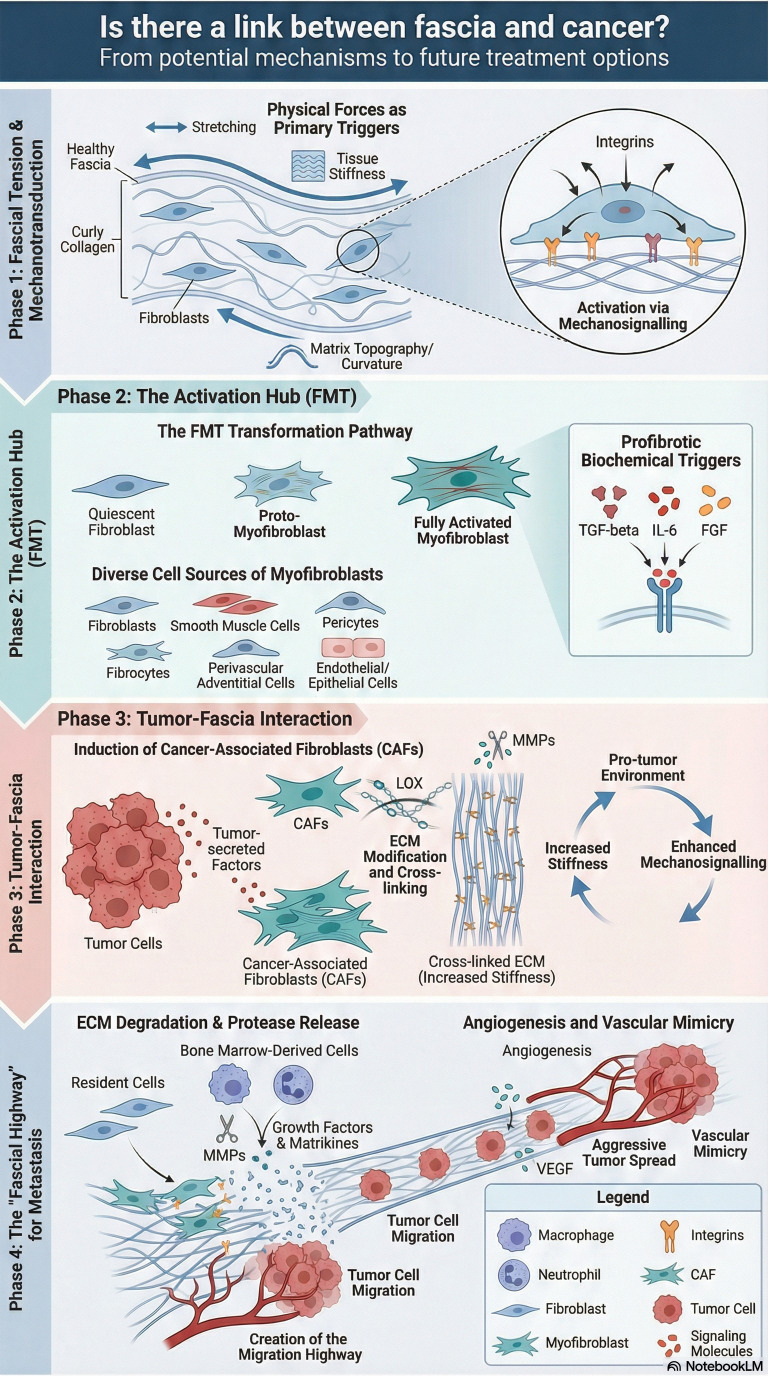
The infographic “Is there a link between fascia and cancer?” (designed with the aid of Google NotebookLM, Google LLC, 2026) is structured into four key conceptual sections that detail the dynamic interaction between fascial tension, fibroblast activation, and the resulting tumor cell response. Note: The figure was created using Google NotebookLM [“graphical abstract synthesized from uploaded source documents”]; Google LLC. Available from https://notebooklm.google.com (accessed February 23, 2026).

Phase 1. Fascial tension and mechanotransduction

This section illustrates how the physical environment of fascia influences cellular behavior.

- Mechanical triggers: Physical factors such as tissue topography, stiffness, curvature, and stretching act as primary signals to the resident cells.
- Mechanosignaling: These physical cues are converted into biochemical signals through a process of mechanosignaling, which is further amplified as the extracellular matrix (ECM) becomes increasingly stiff.

Phase 2. The activation hub: Fibroblast-to-myofibroblast transformation (FMT)

This section details the cellular transition at the heart of fascial remodeling.

- Cellular plasticity: Myofibroblasts, the key effectors of fascial tension, can arise from multiple sources, including quiescent fibroblasts, smooth muscle cells, epithelial cells, endothelial cells, and pericytes.
- Biochemical activation: The transformation is driven by profibrotic factors like TGF-β, IL-6, FGF, and PDGF, which can be released by the tumor or by the fibroblasts themselves in an autocrine loop.
- The FMT process: Cells progress from a quiescent fibroblast state through a proto-myofibroblast stage (characterized by stress fibers) to become fully active myofibroblasts that synthesize dense ECM.

Phase 3. The dynamic tumor–fascia interaction

This section focuses on how the tumor “reprograms” the fascia to its advantage.

- CAF recruitment: Tumor-secreted factors induce the activation of stromal cells into cancer-associated fibroblasts (CAFs).
- ECM deposition and modification: These CAFs actively deposit new ECM and facilitate ECM cross-linking (via LOX), which significantly increases ECM stiffness.
- Feedback loop: This increased stiffness creates a vicious cycle, further enhancing mechanosignaling and myofibroblast activity.

Phase 4. The “fascial highway” for metastasis

The final section depicts the structural changes that facilitate cancer progression.

- ECM degradation: Proteases (MMPs) secreted by both resident and recruited cells degrade the ECM, releasing growth factors and matrikines.
- Migration and angiogenesis: While modified ECM can initially act as a migration barrier, the remodeling eventually facilitates tumor cell migration and angiogenesis (the growth of new blood vessels).
- Metastatic spread: This remodeled fascial landscape serves as a “fascial highway,” utilizing vascular mimicry and recruitment of bone marrow-derived cells (BMDCs) to support aggressive tumor growth and systemic spread.

This infographic effectively visualizes how the fascia, rather than being a passive wrapping, is a dynamic participant in cancer progression, where mechanical tension and cellular activation create a supportive environment for tumor growth and metastasis.

Key findings:

- Tumor-induced ECM stiffening, fibrosis, and desmoplasia promote tumor growth, invasion, metastasis, and immune evasion.
- Cancer-associated fibroblasts (CAFs) drive ECM remodeling, collagen overproduction, and stiffness, contributing to a pro-tumor microenvironment.
- Mechanical cues from stiff ECM regulate cancer cell behavior through pathways such as YAP/TAZ and integrin signaling, enhancing invasion and therapy resistance.
- Fascia interacts with immune and nervous systems, affecting immune cell trafficking, inflammation, and systemic stress responses.
- Physical therapies like stretching may reduce fascial stiffness and tumor growth, showing distinct molecular effects compared to other exercise types.
- A feedback loop between fibroblasts, immune cells, and ECM remodeling impacts fibrosis and tumor progression.

Main mechanisms:

- Matrix stiffening arises from collagen accumulation, cross-linking (e.g., lysyl oxidase activity), and cell-mediated contraction within the ECM.
- Cancer cells respond to ECM stiffness in a biphasic manner; intermediate stiffness promotes invasion while too soft or too stiff ECM limits it.
- The tumor microenvironment’s fibrosis induces hypoxia and immune suppression, further favoring tumor progression.
- Tumor cells exploit pre-existing ECM clefts for migration, aided by remodeling collagen fibers.
- Fasciacytes (specialized fascia cells) play roles in hyaluronan production and cancer-promoting activities via proteins such as S100A4.

Clinical relevance:

- Fascia involvement and ECM stiffness can be prognostic indicators of aggressive tumors, recurrence, and poor outcome.
- Targeting ECM components, such as collagen cross-linking, is a promising therapeutic avenue, with LOX inhibitors and other drugs showing preclinical benefit.
- Physical modalities reducing fascial stiffness could improve symptoms and potentially influence tumor behavior in supportive cancer care.
- Understanding fascia’s role is critical in integrative oncology and may guide safer and more effective therapies.

Future perspectives:

- More research is needed on fascial modulation to limit cancer progression and improve therapy resistance.
- Imaging and molecular tools should be advanced to better assess fascia in oncology.
- Clinical trials are needed to validate physical fascial therapies (e.g., stretching, massage) for cancer patients and study systemic effects.
- Integrative, interdisciplinary approaches combining mechanobiology, immunology, and oncology will likely yield innovative treatments.
- Personalized mechano-medicine targeting fascia and ECM stiffness could revolutionize cancer care.

## 1 Introduction

Fascia is a sheet or network of connective tissue found throughout the body, forming a continuous structure that envelops, separates, and supports muscles, organs, nerves, blood vessels, and other internal structures (Bordoni et al., 2025). It is primarily composed of collagen fibers, making it both strong and flexible. Fascia forms multiple layers, usually described as superficial (just under the skin), deep (surrounding muscles, bones, and vessels), and visceral (encasing internal organs) (Bordoni et al., 2025).

Fascia supports, separates, and anchors all body structures, contributing to the overall shape and posture of the body. Muscles contract more efficiently because of their fascial envelopes (Bordoni et al., 2025). It reduces friction between muscles, permitting smooth gliding and efficient movement. Fascia transmits mechanical forces generated by muscle contractions across joints, distributing tension much like a network. This contributes to stability and coordinated motion (Slater et al., 2024).

Fascia is also rich in sensory receptors (for stretch, pressure, and pain), making it sensitive—in some regions, even more than skin or muscle. It is involved in proprioception (body awareness) (Slater et al., 2024), exteroception (external sensing), and interoception (internal bodily awareness) (Slater et al., 2024). Fascia houses many nerve endings and serves as an interface among musculoskeletal, endocrine, and nervous systems. It helps regulate blood and lymph flow, hormone distribution, neurotransmitter diffusion, immune response, temperature, and wound healing (Slater et al., 2024). Similar to tendons, fascia can store and release elastic energy (important in movements like jumping and sprinting), making locomotion more efficient. Fascia acts as a barrier to infection and the spread of disease by compartmentalizing structures. It protects underlying nerves, vessels, and organs from mechanical damage. Fascia remodels itself in response to mechanical stress, posture, movement, hydration, and other factors, evolving throughout life to meet functional demands (Slater et al., 2024).

Fascia is no longer seen as a mere passive wrapping; it is a dynamic, adaptive “network” that supports, connects, protects, senses, and integrates the body’s diverse systems into a functional whole (Bordoni et al., 2025). Disorders in fascial health can result in chronic pain, restricted motion, and functional deficits throughout the body. There is growing scientific recognition of fascia—not merely as a passive connective tissue, but as an active and dynamic participant in cancer biology and oncology.

Recent research has shifted understanding in several significant ways: Fascia, as a component of the connective tissue matrix, plays a crucial role in the architecture and mechanics of the tumor microenvironment (TME). Increased tissue stiffness and inflammation in fascia are now seen as driving factors in tumor growth and spread. Dense or fibrotic fascia can facilitate cancer cell invasion and metastatic behavior, while also influencing the immune landscape around tumors (Langevin et al., 2016). Fascia is rich in cellular signaling components. Aberrant signaling through fascia—particularly due to chronic inflammation and altered biomechanical stress—has been implicated in cancer initiation and progression. New studies suggest that fascia’s regulatory role in inflammation may represent both a risk and a potential therapeutic target for cancer management (Langevin et al., 2016; Slater et al., 2024). Fascia interacts closely with the immune and nervous systems. It mediates immune cell trafficking, modulates neuroinflammatory responses, and is involved in broader systemic stress responses via hormones and neurotransmitters. Such properties suggest its involvement in how the body copes with or resists malignancy and therapies (Slater et al., 2024).

Physical-based therapies such as massage, yoga, and acupuncture—often used as part of integrative oncology—may exert beneficial effects by reducing fascial stiffness, inflammation, and fibrosis. This could not only yield symptomatic benefits (e.g., pain relief, improved mobility) but potentially influence cancer progression at the tissue level. However, caution is advised, as the effects of directly applying mechanical forces near tumors are not entirely understood.

The fascial system’s newly recognized roles demand further research, particularly regarding:

- How fascial modulation could potentially limit cancer progression or recurrence.
- Safe inclusion of fascial-targeted therapies in supportive cancer care.
- The development of imaging and molecular tools to better map fascia’s involvement in oncology.

Fascia is moving from a background player to a central, dynamic field of study in oncology, with relevance in cancer biology, progression, immune response, therapy, and overall patient quality of life. This emerging recognition opens up new avenues for research, treatment, and possibly prevention strategies in the future (Slater et al., 2024; Langevin et al., 2016).

With this article, we aim to critically review and synthesize current evidence on the link between fascia, connective tissue remodeling, and cancer biology, with a particular focus on the TME. We also aim to elucidate the cellular and molecular mechanisms by which fascia and extracellular matrix (ECM) stiffening contribute to cancer progression, metastasis, immune evasion, and therapy resistance. Another goal is to explore the clinical relevance of fibrosis, fascia infiltration, and tissue stiffness as prognostic indicators in cancer, highlighting their impact on treatment outcomes. We discuss potential therapeutic strategies targeting fascia and the mechanical properties of the TME, including pharmacological, mechanobiological, and physical interventions. Lastly, we want to identify key gaps in knowledge and outline priorities for future research, particularly regarding the modulation of the fibrotic microenvironment to improve cancer treatment and patient survival.

## 2 The connective tissue network: fascia and ECM in health and cancer

### 2.1 Definition of fascia

The fascial system consists of the three-dimensional continuum of soft, collagen-containing, loose and dense fibrous connective tissues that permeate the body. It incorporates elements such as adipose tissue, adventitia, and neurovascular sheaths, aponeuroses, deep and superficial fasciae, epineurium, joint capsules, ligaments, membranes, meninges, myofascial expansions, periosteum, retinacula, septa, tendons, visceral fasciae, and all of the intramuscular and intermuscular connective tissues including endo-/peri-/epimysium. The fascial system interpenetrates and surrounds all organs, muscles, bones, and nerve fibers, endowing the body with a functional structure and providing an environment that enables all body systems to operate in an integrated manner (Adstrum et al., 2017).

What makes fascia distinct from general tumor stroma?

While fascia shares compositional elements with the broader ECM—including fibrillar collagen, glycosaminoglycans, and embedded fibroblasts (Slater et al., 2024; Bordoni et al., 2025)—it constitutes a functionally and anatomically distinct entity in cancer biology. Three specific properties differentiate fascial contributions from general stromal mechanotransduction.

First is the fasciacyte population. Fasciacytes, first characterized by Stecco and colleagues in 2018, represent a previously unrecognized class of fibroblast-like cells uniquely positioned at the boundaries between fascial sublayers (Stecco et al., 2018; Fede et al., 2021). Unlike conventional fibroblasts that respond primarily to tensile load, fasciacytes are specifically activated by shear forces (Stecco et al., 2018). Their molecular signature, co-expression of S100A4, hyaluronan synthase 2 (HAS2), and vimentin, with the absence of CD68, is unique to this cell type and functionally tied to their role in facilitating inter-layer fascial sliding through the secretion of high-molecular-weight hyaluronan (Wang et al., 2023; Fede et al., 2021). In pathological settings, S100A4 overexpression in fasciacytes and surrounding stroma has been linked to cancer cell motility and invasion (Wang et al., 2023), a cancer-promoting activity with no direct analogue in general tumor stroma.

Second are the macroscopic anatomical invasion corridors. Fascial planes provide macroscopic anatomical pathways, aligned collagen architecture extending over centimeter-length scales, that cancer cells exploit as dedicated highways for perineural and perivascular invasion (Fang et al., 2014; Li et al., 2017; Miyazaki et al., 2019). This structural feature allows metastatic cells to travel along fascial planes over considerable distances with relatively low resistance, a route increasingly recognized in surgical oncology as a primary mechanism of regional spread (Lee et al., 2020). The creation of these aligned collagen tracks is orchestrated by CAF-mediated MMP secretion and fiber reorganization and is distinct from random interstitial matrix penetration described in general ECM biology (Fang et al., 2014; Li et al., 2017).

Third is the independent prognostic value of fascial infiltration. Fascial involvement carries independent prognostic significance beyond what is captured by tumor depth or generic ECM stiffness metrics. In soft tissue sarcomas, tumor invasion of fascial layers is strongly associated with increased local recurrence and reduced disease-free survival (Pasquali et al., 2018; Lee et al., 2020). Critically, clinical data indicate that fascial infiltration may be a stronger predictor of poor prognosis than tumor depth alone (Pasquali et al., 2018; Lee et al., 2020), a pathological variable evaluated in surgical margins that has no equivalent in pure ECM biology.

These three distinctions—a unique shear-responsive cell type, anatomically defined metastatic corridors, and an independent prognostic surgical variable—together justify a fascia-centric conceptual framework rather than a mere reframing of established ECM/TME biology.

### 2.2 The role of the ECM in tissue homeostasis

The ECM plays a crucial role in tissue homeostasis by providing both structural support and dynamic regulatory functions. It forms a complex, three-dimensional scaffold around cells that maintains tissue shape, flexibility, and mechanical properties such as tensile strength and compression buffering. Beyond physical support, the ECM also regulates cellular behavior by modulating cell proliferation, differentiation, migration, and signaling through cell receptors like integrins. It achieves this by providing biochemical cues, sequestering growth factors, and responding to mechanical stimuli such as stiffness and tension.

ECM homeostasis is tightly controlled through a continuous balance of synthesis, modification, and enzymatic degradation of ECM components. This dynamic remodeling is essential for normal development, tissue repair, and organ function. Disruption of ECM homeostasis can lead to pathological conditions including fibrosis, cancer progression, and impaired tissue regeneration, phenomena that are particularly evident in disorders like graft-versus-host disease (GVHD). GVHD fundamentally alters tissue repair by targeting the very cells and mechanisms responsible for regeneration, resulting in significant clinical morbidity and ongoing research into regenerative therapies and immunomodulatory strategies (Ara and Hashimoto, 2021; Link-Rachner et al., 2022; Chakraverty and Teshima, 2021).

In specific tissues such as muscle and heart, ECM remodeling regulates stem cell niches and cellular communication critically for maintaining function and responding to injury.

In summary, the ECM maintains tissue homeostasis through:

- Providing structural and mechanical support to tissues.
- Regulating cell behavior via biochemical and biomechanical signals.
- Sequestering and releasing growth factors to coordinate cellular responses.
- Dynamically remodeling to adapt to developmental, physiological, and pathological demands.

This intricate balance ensures proper tissue morphogenesis, differentiation, and maintenance of organ function during health and repair processes (Cox and Erler, 2011; Frantz et al., 2010; Silva et al., 2020; Zhang et al., 2021; Wang et al., 2025b).

### 2.3 Collagen diversity and abundance in tissues

The human body develops from a single cell that divides into trillions, all sharing the same genetic material. Cell specialization and organ formation are driven by the ECM, which provides the necessary physical and biochemical context for cells to interact, behave appropriately, and build the complex structures of the body. This remarkable process is orchestrated not just by genes but by the context in which cells exist, the ECM. The ECM is a non-cellular dynamic three-dimensional network that provides stability, signaling, and structural support to the organs. The structural backbone of the ECM constitutes collagen, the most abundant protein in the ECM, providing structural integrity to tissues serving as the critical component that provides context for cellular behavior and organ formation (Kular et al., 2014). Different collagen types serve specialized functions across various organs, reflecting the remarkable diversity of ECM composition. Its abundance varies greatly between different organs and tissues in wild-type CD1 female mice (0.1% in brain and liver, 1% in heart and kidney, 3.5% in muscle and lung, 5.7% in colon, 20% in skin, 25%–35% in bone, and approximately 40% in tendon) (Tarnutzer et al., 2023).

For example, type I collagen, the most abundant fibrillar collagen, creates strong fibers that provide tensile strength and structural support to organs, tendons, and bones (Hudson et al., 2021; Amirrah et al., 2022; Gouissem et al., 2022). In contrast, type IV collagen forms basement membranes and serves as the first line of defense, maintaining barriers between tissue compartments while enabling crucial cell signaling by interacting with other proteins (Boudko et al., 2018; Mao et al., 2015; Mak and Mei, 2017; Pokidysheva et al., 2025). This organ-specific distribution reflects the unique mechanical and functional requirements of different tissues, with the heart’s ECM composed primarily of type I and III collagens that provide essential structural support for cardiomyocytes (Marijianowski et al., 1995; Wei et al., 1999; López et al., 2015). In the lungs specifically, type IV collagen in the alveoli membrane facilitates oxygen exchange into the blood, demonstrating how collagen structure directly supports organ function (Lo et al., 2015; Mereness and Mariani, 2021). The basement membrane composition and organization are critical for maintaining proper gas exchange and lung architecture (Wells, 2022; Jones et al., 2018). The interstitial matrix’s collagens swell as chronic conditions like lung fibrosis progress. The patient may experience a reduction in lung function that could be fatal as a result of the tissue becoming rigid. Most chronic diseases, including cancer and cardiovascular disorders, exhibit alterations in collagens in addition to lung diseases (Karsdal et al., 2021).

### 2.4 Fascia as an interface between structure and function

Fascia acts as an essential interface between structure and function in the human body by forming a continuous, dynamic connective tissue network that links and integrates all anatomical components—from muscles, bones, and organs to nerves and blood vessels. This integrative web not only imparts mechanical strength and flexibility but also mediates crucial physiological processes that underlies movement, stability, sensory perception, and tissue health.

Fascia’s unique ability to both support structure and facilitate function makes it a key mediator of health and performance. Modern research recognizes fascia as a dynamic, all-encompassing tissue that not only holds our bodies together but also enables coordination, adaptability, and integrated physiological activity (Bordoni et al., 2025; Slater et al., 2024; Armstrong, 2021).

## 3 Mechanisms linking fascia, ECM, and cancer progression

### 3.1 Tumor-induced stiffening and matrix remodeling

#### 3.1.1 Matrix stiffening: contribution of collagen, cross-linking, and cell-mediated contraction

Research has shown that matrix stiffening is caused by the accumulation, contraction, and cross-linking of the ECM by cancer and stromal cells (Ishihara and Haga, 2022; Zhang and Zhang, 2025). Cancer and stromal cells respond to matrix stiffness, which determines the phenotypes of these cells (Ishihara and Haga, 2022). Increased matrix rigidity associated with the fibrotic reaction is documented to stimulate intracellular signaling pathways that promote cancer cell survival and tumor growth. Pancreatic cancer, for example, is one of the stiffest of all human solid tumors (Rice et al., 2017). In addition, matrix stiffness activates and/or inactivates specific transcription factors in cancer and stromal cells to regulate cancer progression. Several mechanisms of cancer stiffening and progression are regulated by transcription factors responding to matrix stiffness (Ishihara and Haga, 2022).

Changes in mechanosensing integrins have also been discussed: increase in ECM stiffness in and around tumors can lead to increased integrin clustering, resulting in enhanced mechanotransduction, which may then promote cell migration (Paszek and Weaver, 2004; Baker and Zaman, 2010).

#### 3.1.2 Fascial densification and the YAP/TAZ mechanosignaling axis: from stiffness to EMT

**Figure 1:**
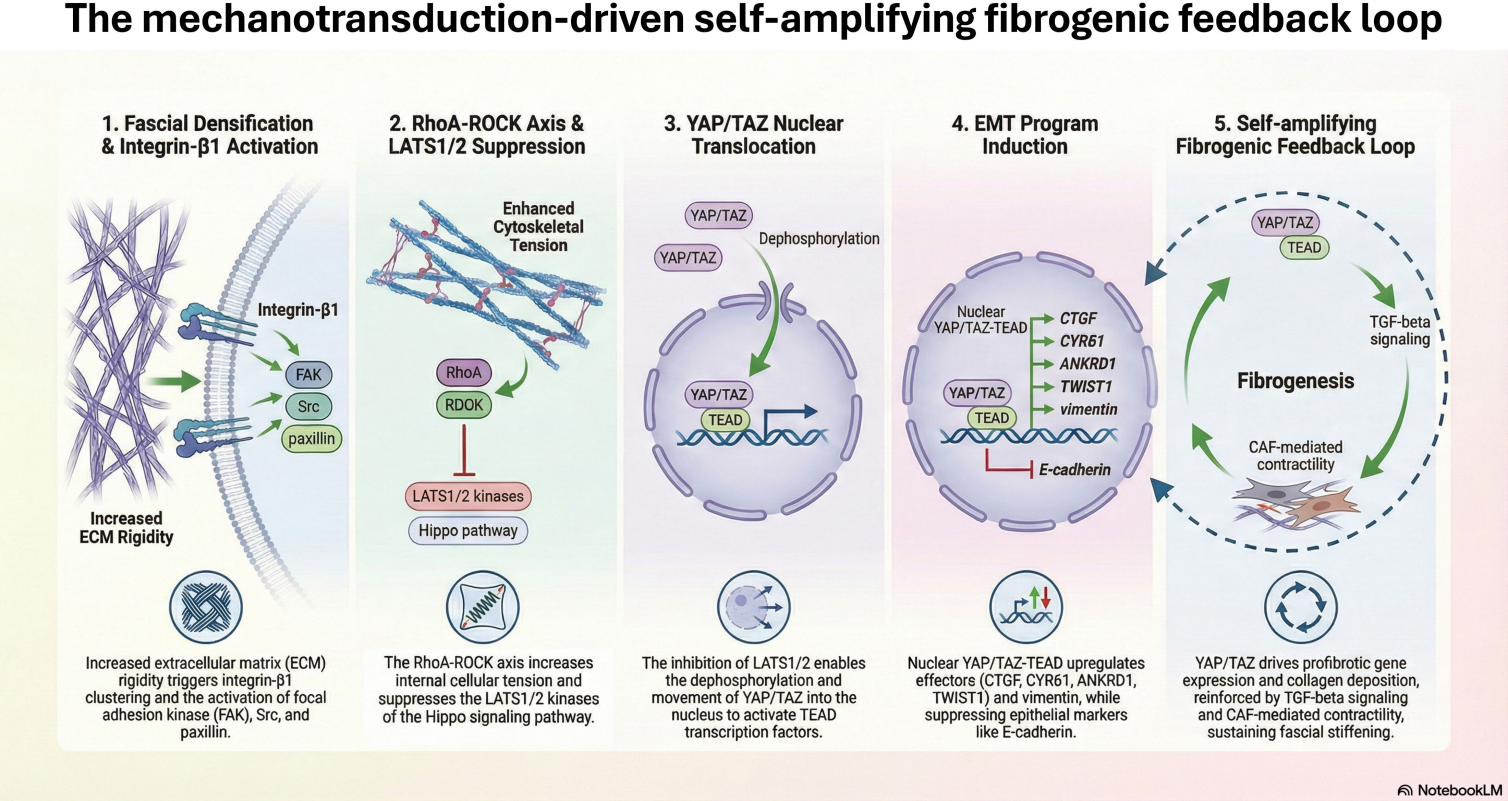
The mechanotransduction-driven self-amplifying fibrogenic feedback loop (designed with the aid of Google NotebookLM, Google LLC, 2026). The process of fibrogenesis is initiated by increased extracellular matrix (ECM) rigidity, which triggers integrin-β1 clustering and the subsequent activation of focal adhesion kinase (FAK), Src, and paxillin. (1) Fascial densification leads to FAK-mediated activation of the RhoA–ROCK–actomyosin axis, which enhances cytoskeletal tension and suppresses the LATS1/2 kinases of the Hippo pathway. (2) The inhibition of LATS1/2 enables the dephosphorylation and nuclear translocation of YAP/TAZ, allowing it to activate TEAD transcription factors. (3) The resulting nuclear YAP/TAZ–TEAD complex induces an epithelial–mesenchymal transition (EMT) program by upregulating profibrotic effectors—including CTGF, CYR61, ANKRD1, TWIST1, and vimentin—while suppressing epithelial markers such as E-cadherin. (4) This signaling cascade culminates in a self-amplifying fibrogenic feedback loop, where YAP/TAZ-driven gene expression and collagen deposition are reinforced by TGF-beta signaling and cancer-associated fibroblast (CAF)-mediated contractility, sustaining fascial stiffening and promoting disease progression.

Fascial densification, the progressive stiffening of the normally pliable inter-sublayer connective tissue through hyaluronan aggregation, collagen fiber thickening, and loss of sliding capacity, constitutes a mechanobiological distinct upstream trigger for YAP/TAZ activation in the peri-tumoral environment. The signaling cascade operates through the following sequential steps, each supported by experimental evidence (**Figure 1**).

Step 1: Integrin clustering and FAK activation: Increased ECM rigidity, such as that generated by fascial densification or CAF-mediated collagen deposition, promotes clustering of integrin-β1 heterodimers at the cell surface. This leads to phosphorylation and activation of focal adhesion kinase (FAK) and its downstream partners Src and paxillin (Paszek and Weaver, 2004; Baker and Zaman, 2010; Ishihara and Haga, 2022).

Step 2: RhoA–ROCK–actomyosin axis: Activated FAK stimulates Rho GTPase (RhoA), which, via Rho-associated protein kinase (ROCK), drives actin polymerization and actomyosin contractility (Doolin et al., 2021; Chang et al., 2025). This elevated cytoskeletal tension physically suppresses the LATS1/2 kinases of the Hippo pathway (Dupont et al., 2011).

Step 3: Nuclear translocation of YAP/TAZ: LATS1/2 suppression leads to dephosphorylation of YAP (Yes-associated protein) and TAZ (transcriptional co-activator with PDZ-binding motif) and their consequent nuclear translocation, where they function as co-activators of TEAD transcription factors (Dupont et al., 2011; Di et al., 2023; Panciera et al., 2017). This YAP/TAZ nuclear accumulation has been directly demonstrated in cancer cells cultured on hydrogel matrices replicating tumor stiffness ranges, confirming the causal sufficiency of mechanical input to drive this step (Dupont et al., 2011).

Step 4: YAP/TAZ-driven EMT gene program: Nuclear YAP/TAZ upregulates target genes including connective tissue growth factor (CTGF/CCN2), cysteine-rich angiogenic inducer 61 (CYR61), and ANKRD1 (Panciera et al., 2017). Concurrently, matrix stiffness drives the nuclear translocation of TWIST1, an essential mechano-mediator of EMT, by releasing it from its cytoplasmic anchor G3BP2; loss of G3BP2 constitutively activates TWIST1 nuclear localization and synergizes with stiffness to promote invasion and metastasis (Wei et al., 2015). A complementary stiffness-driven pathway involving EPHA2/LYN kinase complex activation leads to TWIST1 nuclear localization and triggers EMT in breast cancer models (Fattet et al., 2020). These mechanotransduction pathways together suppress epithelial identity genes (including E-cadherin, suppressed via miR-200 family repression) while inducing mesenchymal markers including vimentin, fibronectin, and ZEB1/2 (Di et al., 2023; Mierke, 2024; Zhang et al., 2025).

Step 5: Self-amplifying fibrogenic feed-forward loop in fascial fibroblasts and CAFs: Nuclear YAP/TAZ activation in fascial fibroblasts and CAFs does not terminate the cascade but generates a self-reinforcing positive feedback loop that sustains peri-tumoral stiffening. YAP/TAZ directly transactivates profibrotic effectors, including connective tissue growth factor (CTGF/CCN2) and plasminogen activator inhibitor-1 (PAI-1/SERPINE1), which promote collagen deposition and inhibit ECM degradation, respectively (Noguchi et al., 2018; Liu et al., 2015). This was first demonstrated in lung fibroblasts cultured on pathologically stiff matrices (~10–40 kPa, mimicking IPF or tumor desmoplasia), where YAP/TAZ knockdown abolished TGF-β-induced CTGF/PAI-1 upregulation and myofibroblast differentiation (Liu et al., 2015). Constitutively active TAZ overexpressed in fibroblasts on soft matrices (~1 kPa) induced only partial CTGF/PAI-1 expression; full ECM gene programs (collagens I/III, fibronectin) required concurrent stiff matrix input, confirming the mechano-dependency of YAP/TAZ fibrogenic output (Liu et al., 2015).

In the fascial/tumoral context, this loop operates as follows: tumor-derived TGF-β activates YAP/TAZ in fasciacytes and resident fibroblasts → YAP/TAZ–TEAD transactivation of CTGF/PAI-1 → enhanced collagen synthesis (via CTGF) and reduced MMP-mediated degradation (via PAI-1) → fascial densification → amplified mechanical input to YAP/TAZ → further CTGF/PAI-1 secretion (Noguchi et al., 2018; Chang et al., 2025). CAFs amplify this in the TME: YAP/TAZ drives myosin light chain 2 (MLC2) phosphorylation, generating actomyosin contractility that physically remodels collagen fibers into aligned invasion tracks while secreting additional LOX/LOXL2 for cross-linking (Calvo et al., 2013; Kirschmann et al., 2002; Erler and Weaver, 2009). This CAF-specific YAP/TAZ–MLC2 axis was validated in mammary carcinoma models, where YAP knockdown in CAFs reduced matrix contraction, tumor cell invasion, and angiogenesis (Calvo et al., 2013).

The loop’s relevance to fascia-oncology is evidenced by elevated nuclear YAP/TAZ in IPF fibroblastic foci (Liu et al., 2015) and tumor stroma (Noguchi et al., 2018), where it perpetuates a pro-invasive niche long after initial tumor stimuli. YAP/TAZ inhibition (e.g., verteporfin disrupting YAP–TEAD interaction) blocks HSC-to-myofibroblast differentiation and reduces fibrosis in CCl_4_ liver models (Noguchi et al., 2018), suggesting therapeutic potential. Critically, this feed-forward mechanism links fascial stiffening not merely as a passive barrier but as an active, self-sustaining driver of tumor plasticity and therapy resistance, independent of canonical Hippo signaling (Dupont et al., 2011; Di et al., 2023).

The impact on immune response: Stiff ECM physically impedes T-cell infiltration and reduces their proliferation and cytotoxic activity, compromising the effectiveness of immunotherapy and contributing to poorer outcomes (Yui and Oudin, 2024; Micalet et al., 2023; Mai et al., 2024).

Fascia involvement is a critical factor in tumor progression and prognosis, particularly in compliant tissue sarcomas and other malignancies that interact with connective tissue. At least tumor infiltration of fascia is strongly associated with a higher risk of recurrence and a worse prognosis.

When tumors invade or cross fascial layers, this often reflects more aggressive biological behavior and a greater likelihood of local recurrence or metastasis (Lee et al., 2020). Clinical data suggest that fascia involvement may be a better predictor of poor prognosis than tumor depth alone (Pasquali et al., 2018).

Connective tissue biology: Fascia is part of the connective tissue network. Increased local stiffness and collagen alignment at tumor boundaries have been associated with greater invasiveness and progression in both animal models and human biopsies (Langevin et al., 2016). The desmoplastic response—characterized by fibroblast activation, ECM deposition, and stiffness—promotes epithelial–mesenchymal transition (EMT) and tumor invasion (Langevin et al., 2016; Mancini et al., 2024).

**Table 1** gives an overview of the relationship between connective tissue stiffness and cancer. 

Connective tissue and fascia-related stiffness are strongly associated with increased cancer mortality due to their role in promoting tumor progression, metastasis, immune evasion, and therapy resistance. Targeting ECM stiffness and fascia involvement may offer new therapeutic opportunities to improve outcomes in aggressive cancers (Yui and Oudin, 2024; Pasquali et al., 2018; Quintela-Fandino et al., 2024) (**Table 1**).

**Table 1:** Summary: Connective tissue stiffness and cancer.

Factor	Impact on cancer mortality	Mechanism/evidence
ECM/connective tissue stiffness	Increases mortality, metastasis, resistance	Promotes proliferation, invasion, immune evasion (Yui and Oudin, 2024; Ishihara and Haga, 2022; Mai et al., 2024)
Fascia infiltration	Increases mortality, recurrence	Poorer survival in soft tissue sarcoma (Pasquali et al., 2018)
Fibrosis	Adverse prognosis, metastasis	Clinical study in breast cancer (Quintela-Fandino et al., 2024; Mancini et al., 2024)

Research has shown that matrix stiffening is caused by the accumulation, contraction, and cross-linking of the ECM by cancer and stromal cells (Ishihara and Haga, 2022; Zhang and Zhang, 2025). Cancer and stromal cells respond to matrix stiffness, which determines the phenotypes of these cells (Ishihara and Haga, 2022). Increased matrix rigidity associated with the fibrotic reaction is documented to stimulate intracellular signaling pathways that promote cancer cell survival and tumor growth. Pancreatic cancer, for example, is one of the stiffest of all human solid tumors (Rice et al., 2017). In addition, matrix stiffness activates and/or inactivates specific transcription factors in cancer and stromal cells to regulate cancer progression. Several mechanisms of cancer stiffening and progression are regulated by transcription factors responding to matrix stiffness (Ishihara and Haga, 2022).

Changes in mechanosensing integrins have also been discussed: Increase in ECM stiffness in and around tumors can lead to increased integrin clustering, resulting in enhanced mechanotransduction, which may then promote cell migration (Paszek and Weaver, 2004; Baker and Zaman, 2010).

Tumor stiffness arises from:

- ECM accumulation: Overproduction of fibrillar collagens and glycosaminoglycans (e.g., hyaluronan) by cancer-associated fibroblasts (CAFs).
- Collagen cross-linking: Enzymatic activity (e.g., lysyl oxidase) increases ECM rigidity by forming covalent crosslinks. Non-enzymatic cross-linking in ECM (e.g., advanced glycation end products—AGEs) has also been linked to carcinogenesis, especially in the context of aging (Northey and Weaver, 2023; Rossi et al., 2023; Schröter and Höhn, 2018; Haque et al., 2019; Rojas et al., 2018).
- Altered MMP profiles may also play a role in the progressive destruction of normal ECM and replacement of stiffer tumor-derived ECM; they can also impact inflammation and angiogenesis (Jodele et al., 2006; Strongin, 2006; Cox and Erler, 2011).
- LOX activity may furthermore promote invasiveness due to altered three-dimensional architecture (“LOX-enriched leading edge”), potentially leading to increased cell-matrix adhesion, further exacerbating the process of matrix remodeling (Kirschmann et al., 2002; Erler and Weaver, 2009; Payne et al., 2005). Notably, in pancreatic ductal adenocarcinoma, tumor cells and stromal fibroblasts cooperate synergistically via a MT1-MMP/MMP2 axis, whereby MT1-MMP on tumor cells activates fibroblast-secreted pro-MMP2, to drive invadopodia-based ECM degradation and invasion (Cao et al., 2021).
- Cell-mediated contraction: Stromal cells contract the ECM via actin–myosin forces, further stiffening the microenvironment (Sun et al., 2016).

Connective tissue and fascia-related stiffness have significant implications for cancer progression and mortality, primarily through their influence on the TME and ECM mechanics.

#### 3.1.3 Clinical relevance: fibrosis as a prognostic indicator

Fibrosis (excessive ECM stiffness) in tumors is associated with worse prognosis and increased risk of metastasis, as shown in clinical studies, such as those in HER2-negative breast cancer (Quintela-Fandino et al., 2024; Mancini et al., 2024). Therapies aimed at reducing ECM stiffness, such as LOX inhibitors targeting collagen cross-linking, have shown promise in preclinical models to improve drug delivery and reduce metastasis (Yui and Oudin, 2024; Feng et al., 2024a).

The understanding of collagen accumulation and organization as primary stiffness determinants has direct therapeutic implications. Targeting specific components of this system offers potential interventions for fibrotic diseases. LOXL2 inhibition shows promise in reducing established fibrosis and promoting tissue remodeling. Similarly, broad-spectrum LOX inhibition with agents like β-aminopropionitrile (BAPN) can interrupt pathological cross-linking processes (Zhang et al., 2022).

Tissue-specific responses highlight the complexity of collagen mechanics. Different organs exhibit distinct relationships between collagen accumulation, organization, and stiffness. In lung fibrosis, LOXL1 and LOXL2 expression correlates with increased collagen fibril thickness and tissue stiffness. In skeletal muscle, collagen organization shows muscle-specific influences on mechanical properties, with alignment being crucial in some muscles while cross-linking dominates in others (Brashear et al., 2022).

The temporal aspects of collagen remodeling also influence stiffness development. Early intervention targeting collagen synthesis pathways may be more effective than attempting to reverse established cross-linked matrices. However, evidence suggests that even mature fibrotic tissues retain some capacity for remodeling when pathological cross-linking is interrupted (Ikenaga et al., 2017).

Collagen accumulation and organization thus represent interconnected processes that fundamentally determine tissue mechanical properties through molecular-level changes in protein structure, cross-linking chemistry, and hierarchical assembly. Understanding these mechanisms provides crucial insights for developing targeted therapies for fibrotic diseases and optimizing tissue engineering approaches.

#### 3.1.4 Distinguishing association from mechanistic causation

It is important to clearly stratify the levels of evidence underlying the mechanistic claims in this review. The association between increased ECM/fascial stiffness, tumor aggressiveness, and poor prognosis is well supported by clinical data across multiple tumor types: fibrosis predicts worse outcomes in HER2-negative breast cancer (Quintela-Fandino et al., 2024; Mancini et al., 2024), fascial infiltration is associated with higher recurrence and poorer prognosis in soft tissue sarcomas (Pasquali et al., 2018; Lee et al., 2020), and elevated tumor stiffness, as in pancreatic ductal adenocarcinoma, one of the stiffest of all human solid tumors, is consistently associated with worse therapeutic response (Rice et al., 2017). These epidemiological and pathological associations are robust.

Demonstrating direct causation—that fascial stiffening mechanistically drives, rather than merely accompanies, cancer progression—requires experimental manipulation. Here the evidence is more limited but increasingly compelling. Mouse studies by Langevin et al. (2016) demonstrated that mechanical modification of fascial architecture altered tumor growth trajectories in subcutaneous tumor models, constituting a direct experimental manipulation. Berrueta et al. (2018a) showed that daily 10-min stretching reduced the tumor volume by 52% in an orthotopic mouse breast cancer model alongside measurable reductions in stromal TGF-β, collagen density, and immune exhaustion markers (Berrueta et al., 2018a). He et al. (2025) confirmed in a voluntary stretching model that fascial mobilization, independent of aerobic locomotion, suppressed tumor growth via distinct plasma proteomic signatures including upregulation of adiponectin (He et al., 2025). *In vitro* experiments using tunable hydrogel matrices of defined stiffness have established direct causal relationships between substrate rigidity, YAP/TAZ nuclear translocation, TWIST1 release from its cytoplasmic anchor G3BP2, EMT marker expression, and cancer cell invasiveness (Dupont et al., 2011; Wei et al., 2015; Fattet et al., 2020), constituting well-controlled mechanistic causation at the cell level.

The weight of evidence supports a model in which fascial stiffening is both a consequence of tumor activity (through CAF-mediated ECM remodeling and TGF-β signaling) and a mechanobiological co-driver of progression, a positive feedback loop rather than unidirectional causation (Zeltz et al., 2020; Chandler et al., 2019).

### 3.2 Desmoplasia and the tumor microenvironment

Desmoplasia is a hallmark of the tumor microenvironment (TME) characterized by the formation of dense fibrous tissue due to the accumulation of extracellular matrix (ECM) components and activated fibroblasts, particularly cancer-associated fibroblasts (CAFs). This process is commonly observed in various solid tumors including pancreatic ductal adenocarcinoma, breast, lung, and cervical cancers.

The TME is a complex network comprising ECM macromolecules and a variety of cells such as CAFs, immune cells, endothelial cells, and pericytes. In desmoplastic tumors, ECM homeostasis is disrupted, leading to excessive fibrillar collagen accumulation and altered matrix stiffness (Zeltz et al., 2020).

CAFs are central to desmoplasia, as they produce ECM proteins and release paracrine signals that influence tumor growth, angiogenesis, metastasis, immune suppression, and resistance to treatment. CAFs also contribute to remodeling the TME by switching fibroblasts to myofibroblasts and altering the ECM architecture (Zeltz et al., 2020).

Desmoplasia creates a physical and biochemical barrier that can promote tumor proliferation, invasion, and metastasis. It often results in hypoxia due to reduced tumor perfusion and increases solid stress on cancer cells. These features promote EMT, aggressive tumor behavior, and resistance to chemotherapy (Zeltz et al., 2020).

The desmoplastic TME commonly fosters immunosuppression, reducing the infiltration and activity of cytotoxic lymphocytes. CAFs and ECM components modulate immune responses, for instance, by secreting factors that skew immune cell populations toward tumor-supportive phenotypes (Wolf et al., 2023).

Because desmoplasia contributes to therapy resistance by limiting drug delivery and fostering immunosuppression, targeting components of the desmoplastic reaction within the TME (such as CAFs or ECM remodeling pathways) is a promising strategy for improving cancer treatment outcomes (Zeltz et al., 2020).

In summary, desmoplasia is a key feature of the TME that profoundly influences tumor biology, immune evasion, and therapeutic resistance through its dense ECM, activated fibroblasts, and altered cellular interactions. Understanding and targeting desmoplasia within the TME is a critical area of cancer research and therapy development.

#### 3.2.1 Role of fibroblast activation and ECM deposition

Fibroblast activation and extracellular matrix (ECM) deposition are essential biological processes in tissue repair and pathology, particularly fibrosis and cancer.

Transition to active state: Fibroblasts, normally quiescent, become activated in response to mechanical stress, injury, or molecular signals (e.g., TGF-β, cytokines). Upon activation, they change morphology, increase proliferation, and become more migratory. This activation often involves the transition to myofibroblasts, which express contractile proteins like α-smooth muscle actin (αSMA).

Functional changes: Activated fibroblasts exhibit increased production, remodeling, and secretion of ECM proteins (e.g., collagens, fibronectin, periostin) as well as enzymes that degrade or modify the matrix (e.g., matrix metalloproteinases).

Dynamic mechanisms: Mechanical cues such as ECM density and matrix stiffness, along with biochemical signals, contribute to activation. This creates feedback loops: activated fibroblasts generate a mechanical microenvironment that perpetuates their activation, especially in chronic conditions like fibrosis and cancer.

Homeostasis and remodeling: ECM is a dynamic structure consisting of proteins like collagen and glycoproteins. Its balanced deposition and degradation maintain tissue homeostasis. Growth factors (e.g., TGF-β, FGF, PDGF) orchestrate the deposition and remodeling of ECM by regulating fibroblast activity.

**Figure 2:**
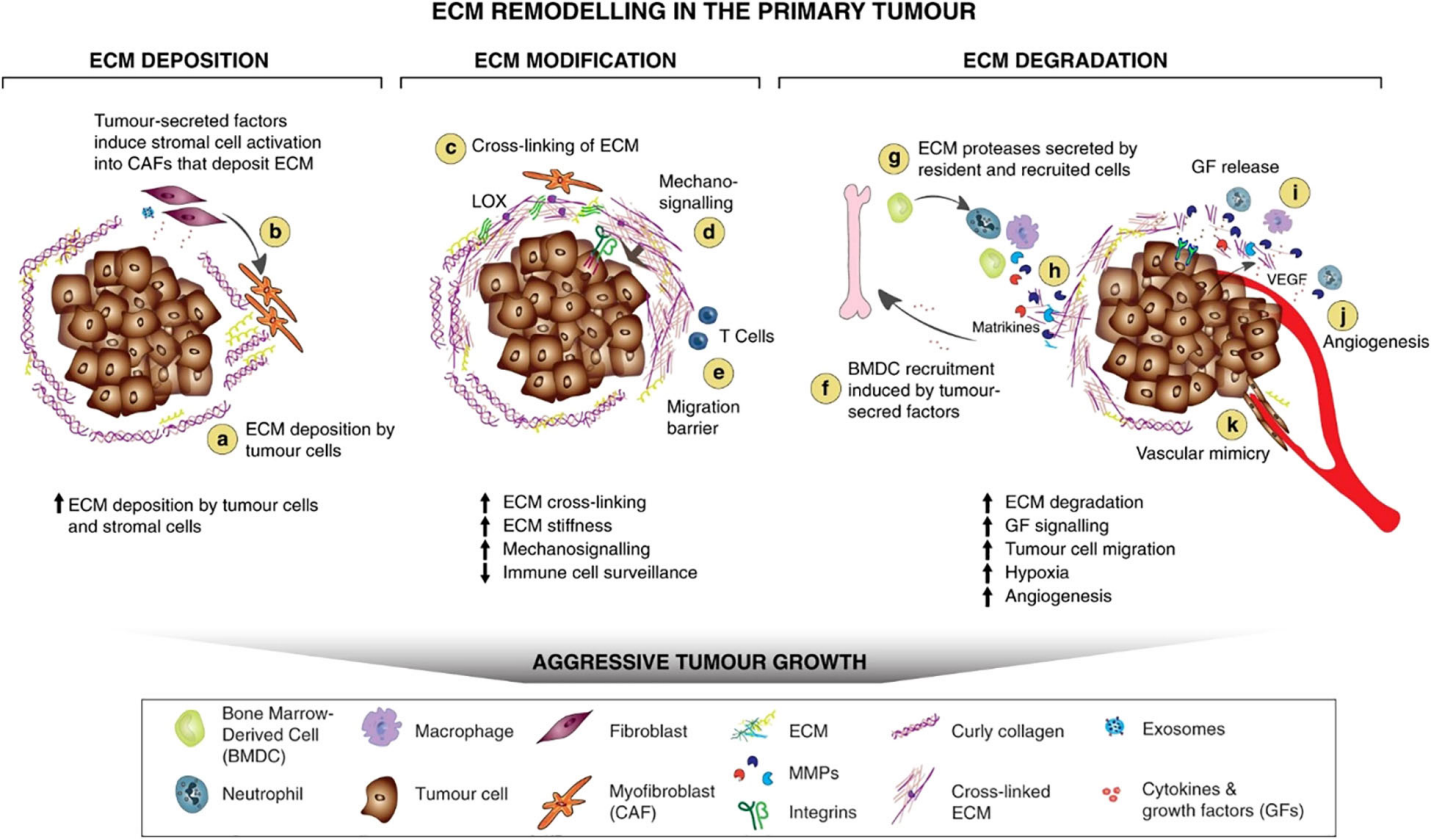
ECM remodeling in the primary tumor (Winkler et al., 2020). Copyright ^©^2020, Winkler et al. This is an open-access article distributed under the terms of the Creative Commons Attribution License (CC BY). **(a, b)** Tumor-derived factors activate stromal cells which differentiate into cancer-associated fibroblasts (CAFs), leading to the secretion and deposition of large amounts of ECM components along with the cancer cells. **(c)** ECM-modifying enzymes such as LOX expressed by tumor cells and CAFs cross-link and align collagen fibers, which increases matrix stiffness around the tumor, and **(e)** the formation of a physical barrier to evade immune surveillance by T cells. **(d)** Increased matrix stiffness promotes the interaction between ECM components and cell-surface receptors on tumor cells that trigger mechanosignaling mediated by integrins. **(f)** To sustain a tumorigenic microenvironment, tumor cells and resident immune cells secrete cytokines, chemokines, and growth factors (GFs), which differentiate and recruit bone-marrow-derived cells (BMDCs). **(g)** The BMDCs, CAFs, and tumor cells secrete ECM-degrading proteases, including MMPs, which are cell-surface-bound (e.g., MT1-MMP) or secreted (e.g., MMP-9). **(h)** Proteolytic ECM degradation generates bioactive matrikines and **(i)** releases matrix-bound GFs. These factors induce pro-tumorigenic ECM signaling that promotes tumor proliferation, migration, invasion, and angiogenesis. **(j)** These combined changes to the ECM create a hypoxic environment. Neutrophils secrete potent MMP-9 that degrades ECM and releases matrix-bound VEGF that forms a concentration gradient for new angiogenic sprouting. **(k)** Stimulated by dense ECM, the tumor cells may gain endothelial-like functions and mimic the vasculature that connects to blood vessels.

**Figure 2** illustrates the mechanisms of extracellular matrix (ECM) remodeling in the primary tumor, highlighting four main processes: (1) ECM deposition, which changes the abundance and composition of ECM components affecting biochemical and mechanical properties, (2) chemical modification at the post-translational level altering the ECM structure, (3) proteolytic degradation that releases bioactive ECM fragments and frees cellular migratory paths, and (4) force-mediated physical remodeling which reorganizes ECM fibers to facilitate cell migration. These dynamic ECM changes, driven by tumor and stromal cells, create a cancer-supporting microenvironment that influences tumor growth, invasion, immune evasion, and vascularization. The figure encapsulates how these remodeling processes contribute to the formation and progression of the primary tumor by modifying both the biochemical signals and the physical structure of the tumor ECM (Winkler et al., 2020).

Fibrosis and disease: Excessive ECM deposition occurs when fibroblast activation is persistent (such as in chronic inflammation or repeated injury). This leads to organ fibrosis, disrupts normal tissue architecture, and impairs organ function. In the heart, for example, pathological ECM accumulation results in cardiac fibrosis and dysfunction.

Bidirectional crosstalk: The ECM does not just provide structural support; it also regulates cell behavior. Changes in ECM composition can promote further fibroblast activation, creating a pathological feedback loop in diseases.

### 3.3 The transition from health to disease

The process is categorized into four distinct stages that transform healthy tissue into a path for tumor spread, namely:

- Normal fascial architecture: In a healthy state, fascia is characterized by sliding fasciacytes and high-molecular weight (HMW) hyaluronan, which facilitates smooth movement between tissue layers.
- Fascial densification: When a tumor releases TGF-β signaling, the fascia loses its ability to slide. It becomes stiff due to the aggregation of hyaluronan and the thickening of collagen fibers.
- CAF-mediated fiber realignment: Cancer-associated fibroblasts (CAFs) then reorganize these thickened collagen fibers into linearized, aligned tracks. These tracks can extend over centimeter-long scales.
- Directional tumor migration: Cancer cells exploit these aligned fibers as “highways” for rapid migration toward perivascular and perineural spaces, allowing the cancer to spread more easily.

#### 3.3.1 Biological mechanisms of remodeling

The source identifies a three-tier system of triggers and mediators that drive this transformation, namely:

- Tier 1 (upstream triggers): Physical factors like fascial densification and increased extracellular matrix (ECM) rigidity, along with tumor-derived TGF-β.
- Tier 2 (intracellular mediators): These include integrin receptor clustering, the RhoA–ROCK axis, and the nuclear translocation of YAP/TAZ, which are key sensors of mechanical stress in cells.
- Tier 3 (effector outputs): These triggers lead to the activation of EMT (epithelial–mesenchymal transition) gene programs and the secretion of profibrotic effectors like CTGF or PAI-1, which sustain a self-amplifying feedback loop of tissue stiffening.

#### 3.3.2 The myofibroblast hub

**Figure 3:**
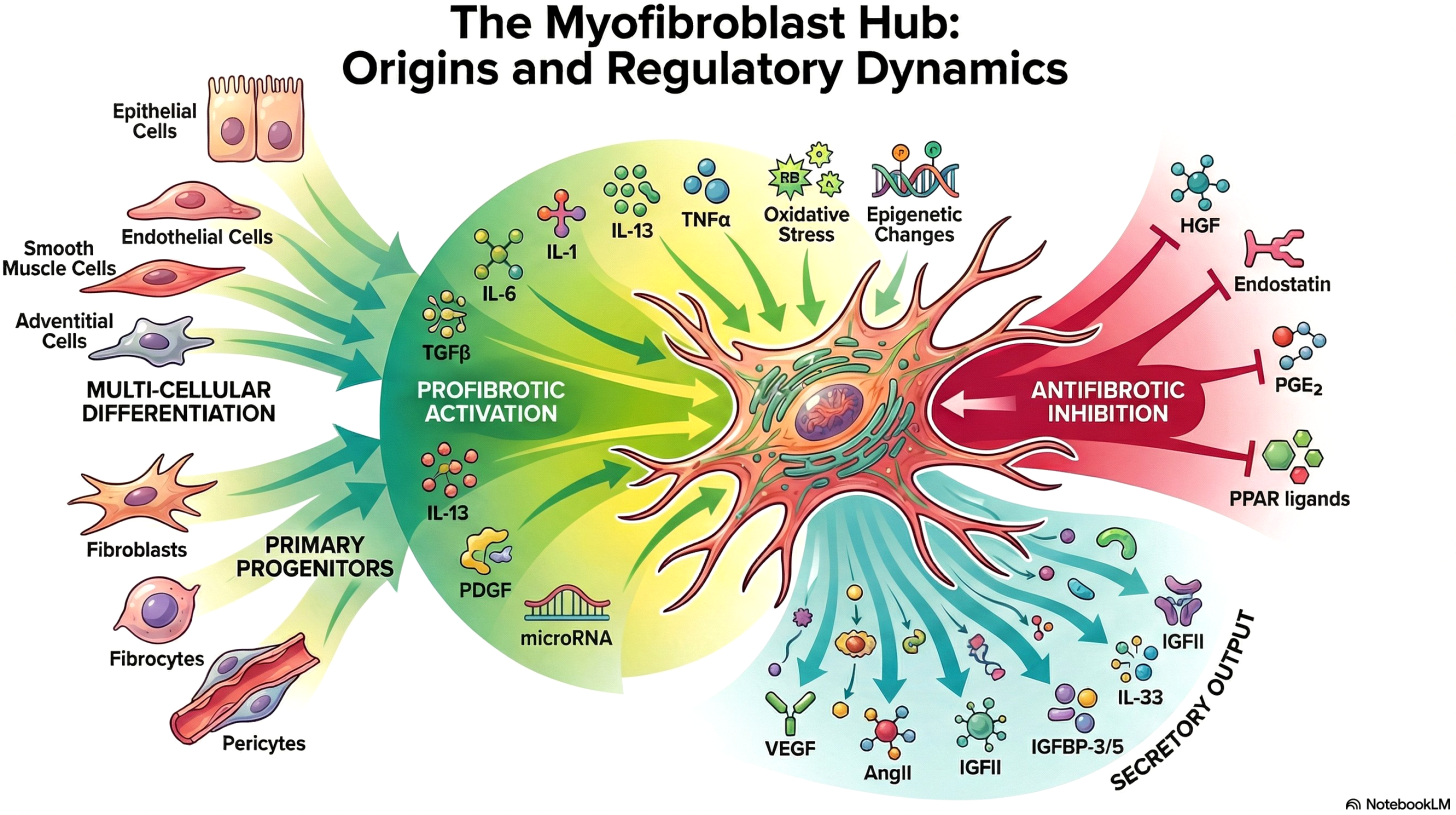
“The Myofibroblast Hub: Origins and Regulatory Dynamics” illustrates myofibroblasts as central orchestrators of connective tissue remodeling, with direct relevance to cancer-associated desmoplasia (designed with the aid of Google NotebookLM, Google LLC, 2026). Myofibroblasts arise from multiple cellular precursors - including fibroblasts, fibrocytes, pericytes, and epithelial, endothelial, smooth muscle, and adventitial cells - via trans-differentiation and multi-cellular differentiation pathways. Profibrotic activation is driven by key cytokines and growth factors (TGF-β, IL-1, IL-6, IL-13, PDGF), microRNA-mediated regulation, and upstream stressors including TNFα, oxidative stress, and epigenetic reprogramming, collectively promoting the emergence of activated α-SMA⁺ myofibroblasts. In the tumor microenvironment (TME), these cells act as central drivers of desmoplastic extracellular matrix (ECM) remodeling through ECM stiffening, collagen alignment, and enhanced protease activity, thereby facilitating cancer cell migration and progression (Tomasek et al., 2002; Kalluri & Zeisberg, 2006). Antifibrotic inhibition is counterbalanced by HGF, Endostatin, PGE₂, and PPAR ligands, which suppress myofibroblast persistence; however, these restraining signals are frequently overridden in established tumor stroma, particularly in pancreatic ductal adenocarcinoma and colorectal cancer (Öhlund et al., 2017; Whatcott et al., 2015). Activated myofibroblasts further shape the TME through paracrine secretory output, including VEGF, Angiotensin II (AngII), IGFII, IGFBP-3/5, and IL-33, promoting angiogenesis, tumor cell proliferation, and immune modulation (Bhowmick et al., 2004). Figure designed with the aid of Google NotebookLM (Google LLC, 2026).

Several cell types contribute to this remodeling process as precursors to the “myofibroblast hub,” including resident fibroblasts, EMT-derived cells, pericytes, fibrocytes from bone marrow, and mesenchymal stem cells (MSCs) (**Figure 3**).

#### 3.3.3 Clinical and therapeutic significance

Understanding the fascial highway has several practical implications for cancer treatment (see **Graphical Abstract**):

- Prognosis: Fascial involvement is a stronger predictor of poor prognosis and recurrence in soft tissue sarcomas than tumor depth alone.
- Drug delivery: Reducing fascial stiffness can decompress intratumoral vessels, which helps normalize blood flow and improves the delivery of chemotherapy and immunotherapy.
- Mechanical intervention: In preclinical models, daily stretching has been observed to reduce tumor volume (by up to approximately 52%), potentially through the modulation of stromal TGF−β signaling and collagen density. However, these findings have not yet been demonstrated in humans, and their clinical relevance remains to be established.

Fibroblast activation and ECM deposition represent fundamental processes in tissue homeostasis, wound healing, and pathological conditions such as fibrosis and cancer. Activated fibroblasts transition from a quiescent state through proto-myofibroblasts to fully contractile myofibroblasts, characterized by α-smooth muscle actin expression and stress fiber formation.

Two principal drivers orchestrate this activation (Chang et al., 2025): On the one hand, TGF-β signaling establishes a positive feedback loop via latent-to-active conversion in the ECM and both SMAD-dependent and SMAD-independent pathways (Zent and Guo, 2018). On the other hand, mechanotransduction through integrin and the FAK–ROCK–MRTF/YAP-TAZ pathway senses matrix stiffness, reinforcing activation and ECM production (Doolin et al., 2021).

Activated fibroblasts are the main source of ECM, producing (see also **Figure 2**) (Winkler et al., 2020) the following:

- Collagens (predominantly type I and III), whose post-translational modifications and LOX-mediated cross-linking determine tissue stiffness (Narayanan et al., 1989).
- Fibronectin fibrils assembled via α5β1 integrin into templates that guide collagen deposition (Garrison and Schwarzbauer, 2021).
- Proteoglycans and hyaluronic acid, regulating hydration and cell migration (Sisson et al., 1980).

The ECM is a crucial component of the TME, especially in solid cancers. In many solid tumors, the ECM can constitute up to 60% of the total tumor mass. This extensive presence highlights its significant influence on tumor biology.

ECM turnover is balanced by matrix metalloproteinases (MMPs) and their inhibitors (TIMPs). Dysregulation—excessive ECM deposition and cross-linking or impaired degradation—drives fibrosis and creates a stiff microenvironment that sustains myofibroblast activation. In cancer, cancer-associated fibroblasts remodel ECM to facilitate tumor invasion and metastasis (Chang et al., 2025).

Therapeutic strategies under investigation include inhibitors of TGF-β signaling, mechanotransduction effectors (YAP/TAZ, ROCK), LOX family enzymes, and MMP/TIMP modulators as well as approaches targeting fibroblast metabolic and mechanical memory (Swaney et al., 2005).

Myofibroblasts can differentiate from a variety of precursor cell types, not just resident fibroblasts. They commonly arise in response to profibrotic cytokine stimulation during injury and fibrosis. Besides resident fibroblasts, myofibroblasts have been shown to derive from epithelial cells, endothelial cells, pericytes, multipotent monocytes, and fibrocytes (circulating fibroblast-like cells from bone marrow). Major developmental signaling pathways, such as Wnt, Notch, and Sonic hedgehog, play key roles in this differentiation. The myofibroblast phenotype is defined by characteristics like αSMA expression, contractility, increased extracellular matrix synthesis, and resistance to apoptosis. These cells are important in wound healing and fibrosis by contracting tissue and remodeling ECM. The exact precursor contribution may differ by tissue type, with some uncertainty remaining.

#### 3.3.4 Cancer-associated fibroblasts, fasciacytes, and myofibroblasts

Tumor-associated fibrosis represents an excessive accumulation of ECM components within and around tumors, primarily produced by myofibroblasts and cancer-associated fibroblasts (CAFs). This fibrotic process critically influences tumor progression by creating physical and biochemical barriers that impact immune surveillance. CAFs play central roles in regulating anti-tumor immunity through direct interactions with immune cells and by modifying the ECM structure (Huang et al., 2020).

CAFs are activated stromal cells within the TME. They secrete large amounts of fibrillar collagens, particularly type I collagen, which increases the density and rigidity of the ECM. This collagen is further stabilized and stiffened through cross-linking enzymes such as lysyl oxidase (LOX), also produced by CAFs (Hanley et al., 2016; Karagiannis et al., 2012). CAFs also produce glycosaminoglycans, especially hyaluronan (HA). Hyaluronan binds to ECM proteins and forms a hydrated, gel-like matrix that increases tissue turgor and stiffness. High levels of hyaluronan are associated with increased interstitial pressure, vascular collapse, hypoxia, and resistance to therapy (Mai et al., 2024; Barkovskaya et al., 2020).

While fascial thickening alters proprioception (e.g., reduced nerve ending density in thoracolumbar fascia), current evidence does not establish inflamed fascia as a root cause of cancer. CAFs primarily originate from local fibroblasts or pericytes—not fascial cells—and their tumor-promoting activities are microenvironment-specific (Mika et al., 2024). According to some studies, myofibroblasts may promote angiogenesis and cancer progression (Liu et al., 2024b). Furthermore, when fascia becomes inflamed, it becomes densified. This thickening can occur in any connective tissue, such as ligaments and tendons, and is problematic because it severely affects proprioception. Chronic fascial inflammation and biomechanical alteration have been identified as contributing microenvironmental factors in certain pathological conditions, including localized pain syndromes, fibrotic disorders, and aspects of cancer progression within the TME (Langevin et al., 2016; Slater et al., 2024; Chandler et al., 2019). However, the available data does not support a primary or singular causal role for fascial pathology in cancer initiation, which remains a multifactorial process governed principally by somatic mutations, oncogene activation, genomic instability, and carcinogenic exposures (Zeltz et al., 2020). Fascial biology constitutes one mechanobiological dimension of the tumor microenvironment and should be framed accordingly. Moreover, while CAF populations overlap with fascial fibroblast lineages, it has been noted that CAFs primarily originate from local fibroblasts or pericytes rather than exclusively from fascial cells, and their tumor-promoting activities are microenvironment-specific (Mika et al., 2024). Research has demonstrated the crucial role of CAFs in promoting tumor growth through their ability to modify the stroma. However, myofibroblasts have been observed to impede angiogenesis, a phenomenon that appears to be paradoxical (Gascard and Tlsty, 2016).

The development of tumor fibrosis also involves complex cellular interactions. Both innate and adaptive immune cells critically regulate myofibroblast activation and fibrogenic responses in various fibrotic diseases (Zhou et al., 2023). Inflammatory responses are orchestrated by activated immune cells, which initiate a series of cellular and molecular processes that result in fibrosis in reaction to external stimuli and microenvironmental factors. Finally, the recruitment and activation of immune cells—including macrophages, neutrophils, natural killer (NK) cells, T cells, and B cells—play a central role in regulating both the progression and regression of fibrogenic development through diverse molecular mechanisms (Zhou et al., 2023) (see **Table 2**).

**Table 2:** Effects of fibrosis on tumors.

Effect	Mechanism
Tumor growth/survival	ECM stiffening, growth factor signaling
Immune evasion	Physical barrier, immunosuppressive signaling
Metastasis/invasion	ECM remodeling, enhanced cell migration
Therapy resistance	Impaired drug delivery, chemoresistance, immunotherapy resistance
Prognostic significance	High fibrosis linked to worse outcomes

A promising avenue for enhancing anticancer treatment outcomes involves the targeted inhibition of fibrosis, particularly through the suppression of TGF-β pathways or the targeting of CAFs. These approaches have the potential to augment drug delivery and bolster immune responses (Chandler et al., 2019). The potential of anti-fibrotic therapies as adjuncts to existing cancer treatments is currently being investigated, with the aim of enhancing efficacy and patient prognosis (Chandler et al., 2019).

CAFs are critical stromal components in tumors, actively shaping the TME through paracrine signaling, ECM remodeling, and interactions with cancer cells (Loh and Ma, 2021). A growing body of evidence indicates a greater understanding of their role in the initiation and progression of tumors (Loh and Ma, 2021). It remains to be mentioned that myofibroblasts, as primary ECM producers, contribute significantly to the development of fibrotic conditions within the TME (Zhou et al., 2023).

CAFs in tumor initiation and invasion (Loh and Ma, 2021):

- Early involvement: CAFs are present from incipient tumor stages, contributing to malignant transformation by secreting oncogenic factors (e.g., TGF-β, IL-6) that enhance cancer stem cell features and EMT.
- ECM remodeling: CAFs deposit collagen, fibronectin, and proteoglycans, creating a stiffened ECM that promotes cancer cell migration and invasion. This mechanical restructuring facilitates metastasis by forming “tracks” for cell movement.
- Paracrine communication: CAFs secrete growth factors (e.g., HGF, FGF) that activate pro-survival pathways (e.g., PI3K/AKT, MAPK) in cancer cells, directly fueling proliferation and therapy resistance.

The relationship between fasciacytes and cancer represents one of the most significant emerging areas in oncological research, bridging the gap between fascial biology and cancer progression. This comprehensive analysis explores the discovery of fasciacytes, their dual roles in normal physiology and cancer pathogenesis, and the therapeutic opportunities they present for modern cancer treatment.

Fasciacytes represent a groundbreaking discovery in fascial research, first identified and characterized by Carla Stecco and colleagues in 2018 (Wang et al., 2023; Fede et al., 2021). These specialized cells constitute a previously unrecognized class of fibroblast-like cells that are fundamentally distinct from traditional fibroblasts in both morphology and function. Unlike the elongated, spindle-shaped appearance of conventional fibroblasts, fasciacytes display a characteristic rounded morphology with prominent nuclei and cytoplasm restricted to the perinuclear region (Fede et al., 2021; Wang et al., 2023). The defining molecular signature of fasciacytes includes several key markers that distinguish them from other connective tissue cells. They express S100A4 protein, a calcium-binding protein associated with chondroid metaplasia, along with hyaluronan synthase 2 (HAS2) and the intermediate filament protein vimentin (Stecco et al., 2018; Wang et al., 2023).

Importantly, fasciacytes are negative for CD68, confirming that they do not originate from the monocyte–macrophage lineage, unlike other hyaluronan-secreting cells such as synoviocytes (Wang et al., 2023; Slater et al., 2024).

These cells are strategically positioned at the borders of fascial sublayers, forming clusters of three to four cells that demarcate the boundaries between loose and fibrous connective tissue (Wang et al., 2023; Fede et al., 2021). This unique anatomical location is crucial to their function, as they serve as the primary source of hyaluronan-rich ECM matrix that facilitates smooth gliding between adjacent fascial layers (Wang et al., 2023; Fede et al., 2021).

In healthy tissues, fasciacytes serve as specialized factories for hyaluronan production, contributing approximately 30% of the total fascial cell population (Slater et al., 2024). Their primary physiological role centers on maintaining the viscoelastic properties of fascial tissue through the synthesis and secretion of high molecular weight hyaluronan (HMW-HA) (Slater et al., 2024; Stecco et al., 2018).

The hyaluronan produced by fasciacytes serves multiple critical functions in normal tissue homeostasis. It acts as a biological lubricant, facilitating smooth sliding between fascial layers during movement and muscle contraction (Slater et al., 2024; Stecco et al., 2018). This lubrication function is essential for preventing fascial adhesions and maintaining optimal tissue mobility (Stecco et al., 2018). Additionally, the hyaluronan-rich matrix provides mechanical support and creates a favorable microenvironment for nerve endings and blood vessels that traverse the fascial layers (Slater et al., 2024; Stecco et al., 2018).

Recent research has revealed that fasciacytes are mechanosensitive cells that respond differently to mechanical stimuli compared to traditional fibroblasts (Stecco et al., 2018). While fibroblasts primarily respond to tensile load and stretch, fasciacytes are specifically activated by shear forces. This differential mechanosensitivity suggests that fasciacytes and fibroblasts work as functional antagonists, with fasciacytes promoting tissue gliding while fibroblasts provide structural integrity (Stecco et al., 2018).

### 3.4 Microcirculation, fascia, and cancer progression

Microcirculation, the network of small blood vessels and capillaries, plays a critical role in cancer biology, especially in metastasis and the tumor microenvironment (TME). Circulating tumor cells (CTCs) interact extensively with microcirculation, where biomechanical forces such as fluid shear stress and vessel constriction influence their survival and metastatic potential. Adaptations by tumor cells to these biomechanical stresses involve cytoskeletal remodeling and activation of signaling pathways like the RhoA–ROCK axis, which regulates cell contractility and motility essential for squeezing through narrow capillaries and surviving vascular transit (Ahmadzadeh et al., 2017; Perea Paizal et al., 2021).

The microcirculation governs not only nutrient and oxygen supply but also drug delivery efficacy in tumors, affected by the often abnormal and chaotic vasculature within the TME (Feng et al., 2024b).

Fascia, as a connective tissue network, closely interacts with and mechanistically influences microcirculatory function. Changes in fascial stiffness and remodeling affect blood flow dynamics, oxygenation, and immune cell trafficking within tumors (Langevin et al., 2016). Fibroblasts in the fascia can transition into cancer-associated fibroblasts (CAFs), which remodel the extracellular matrix (ECM), impact microvascular architecture and tumor stiffness, and facilitate metastatic pathways (Winkler et al., 2020; Zeltz et al., 2020).

This reciprocal relationship between microcirculation and fascia within the TME creates a dynamic environment regulating tumor progression, invasion, and immune infiltration. Fascial remodeling may either enhance or inhibit immune cell migration, influencing the tumor immune contexture and response to therapy (Jiang et al., 2017).

Clinically, interventions targeting the mechanical properties of the TME, such as inhibitors of lysyl oxidase (LOX)-mediated collagen cross-linking, have shown promise in improving drug delivery and reducing metastasis in preclinical studies (Yui and Oudin, 2024). Mechanotherapeutic approaches, including targeted stretching or manual therapies, might improve microvascular function and reduce fascial stiffness, although more clinical trials are needed to validate these effects in cancer patients (Berrueta et al., 2018a; He et al., 2025).

The RhoA–ROCK pathway, central to actomyosin contraction and cellular mechanical response, is a key molecular target under investigation. Inhibition of RhoA–ROCK signaling impairs tumor cell migration and invasiveness in microcirculatory environments, highlighting its therapeutic potential (Ahmadzadeh et al., 2017).

In summary, the integrated regulation of microcirculation and fascia mechanics represents a critical axis in tumor biology, offering novel therapeutic avenues to alter the physical and cellular landscape of the TME and improve cancer treatment outcomes.

### 3.5 Collagen and immune cell interactions

Tumor-induced fibroblasts, often termed cancer-associated fibroblasts (CAFs), become highly active and drive the realignment and remodeling of collagen bundles within the ECM. This leads to a transformation of the collagen network from a curly and loose state to one that is thickened, linearized, and stiffened (Fang et al., 2014; Li et al., 2017; Perentes et al., 2009).

The process is orchestrated by fibroblast secretion of matrix metalloproteinases (MMPs) and other factors that reorganize collagen fibers. These changes promote the creation of aligned collagen tracks or “highways” along which cancer cells—and to a certain extent immune cells—migrate (Fang et al., 2014; Li et al., 2017; Miyazaki et al., 2019).

Experimental imaging and modeling demonstrate that fibroblasts co-align with collagen fibers around tumor clusters, forming long-range ordered patterns that support directional cell motility (Li et al., 2017; Morkunas et al., 2021).

### 3.6 Modes of tumor cell invasion

#### 3.6.1 Guiding immune and tumor cells: clefts in the ECM

The 3D architecture and clefts within the collagenous ECM act as physiological guidewires for cell trafficking. These aligned fibers create low-resistance paths that facilitate migration of both tumor cells and immune cells into and through the TME (Fang et al., 2014; Du et al., 2023).

However, the increased density and cross-linking of collagen in tumors can become detrimental to effective immune response. Dense and linearized collagen poses a physical barrier that hampers later-stage immune cell infiltration into the tumor core and restricts cytotoxic activity, often trapping immune cells in the stroma and impairing tumor eradication (Du et al., 2023).

The distribution, orientation, and density of collagen fibers directly correlate with the abundance and effectiveness of antitumor T cells, with denser collagen associated with fewer and less mobile CD8+ T cells (Du et al., 2023).

#### 3.6.2 EMT and mechanical cues

There is substantial evidence from experimental and review studies showing that mechanical cues play a critical role in regulating EMT. Key findings supporting this include the following:

1. Mechanical stress and MRTF-A regulation: Mechanical stress within epithelial tissues regulates EMT by activating mechanotransduction pathways involving myocardin-related transcription factor A (MRTF-A). Increased mechanical stress promotes Rho GTPase activation, leading to polymerization of the actin cytoskeleton and nuclear translocation of MRTF-A, which enhances the expression of EMT genes and cytoskeleton remodeling. This link was demonstrated in studies where spatial patterns of mechanical stress within mammary epithelial sheets directed where EMT occurred, with MRTF-A nuclear localization marking these high-stress EMT-permissive regions. Disrupting mechanical stress transmission abolished patterned EMT and caused uniform EMT activation (Gjorevski et al., 2012; Gomez et al., 2010).

2. Additional mechanosensitive elements: Ion channels like Piezo1 have been shown to mediate mechanically induced EMT by regulating calcium influx in keratinocytes exposed to mechanical stretch. This highlights multiple mechanoresponsive systems translating mechanical stimuli to biochemical EMT programs (He et al., 2022).

3. Integration of mechanical and biochemical signals: Mechanical cues cooperate with biochemical signals such as transforming growth factor-beta (TGF-β) to spatially pattern EMT within tissues. Mechanical heterogeneity, such as tissue geometry-induced stress gradients, dictates which cells undergo EMT. Biochemical signals alone do not fully explain the spatial patterning of EMT; mechanical forces are necessary for appropriate regulation (Gomez et al., 2010; Scott et al., 2019; Gjorevski et al., 2012).

#### 3.6.3 Role of tissue stiffness in cancer cell motility

ECM stiffness impact on EMT: ECM stiffness is a vital mechanical cue that regulates EMT. Increased matrix stiffness promotes EMT by triggering integrin-mediated mechanosignaling pathways such as FAK/Src and inducing nuclear translocation of transcriptional coactivators like YAP/TAZ. High ECM stiffness supports EMT-related transcription factors including TWIST1, which is released from cytoplasmic anchors and translocates to the nucleus to drive EMT gene expression. These mechanotransduction pathways operate in cancer progression, promoting invasion and metastasis. Experimental data show that matrix stiffening enhances EMT markers and cancer cell invasiveness, and inhibiting stiffness-associated pathways can reduce EMT features (Mierke, 2024; Zhang and Zhang, 2025; Dupont et al., 2011; Wei et al., 2015; Fattet et al., 2020; Gjorevski et al., 2012). In conclusion, the mechanistic evidence includes molecular events linking mechanical stress, ECM stiffness, and mechanotransduction pathways (e.g., involving MRTF-A, Rho GTPases, YAP/TAZ, TWIST1) that induce EMT gene expression and phenotypic transition. Although experimental systems employing tunable stiffness matrices, micro-engineered tissues, and diverse cancer models have consistently linked altered mechanical cues to EMT induction and progression, these models often oversimplify the complex *in vivo* microenvironment. Nonetheless, these findings largely arise from controlled *in vitro* models, and their relevance to the dynamic and heterogeneous *in vivo* microenvironment remains to be fully elucidated. Overall, current evidence supports mechanical signaling as a key regulatory dimension of EMT in developmental, regenerative, and pathological settings.

## 4 Fibrosis, immune evasion, and the tumor microenvironment

### 4.1 Effects of fibrosis on immune function

#### 4.1.1 T cell suppression in fibrotic tumors

Fibrosis creates multiple barriers to effective T cell function within the TME. Fibrosis-induced hypoxia suppresses T cell infiltration and function in tumors through several mechanisms. Constant activation of HIF-1α under hypoxic conditions negatively regulates T cell receptor signal transduction, partially due to increased NF-κB activation (Huang et al., 2020). Additionally, the accumulation of extracellular adenosine within the hypoxic TME triggers immunosuppressive signaling via adenosine receptors (A2AR) on antitumor T cells (Huang et al., 2020).

The physical barriers created by dense fibrotic tissue also directly impede T cell infiltration. Research has demonstrated that tumor-associated fibrosis impairs immune surveillance and T cell-mediated tumor control (Herzog et al., 2023). This barrier effect is particularly significant in cancers with high fibrotic content, which often demonstrate reduced T cell infiltration and poor responses to immune checkpoint inhibitors.

#### 4.1.2 Type 2 immunity in fibrosis

Type 2 immune responses, characterized by the production of IL-4, IL-5, IL-9, and IL-13, significantly contribute to tissue repair and fibrosis following injury (Gieseck et al., 2018). While these responses exhibit host-protective functions, including maintenance of metabolic homeostasis and regulation of tissue regeneration, excessive and chronic activation can lead to pathological fibrosis (Gieseck et al., 2018).

Type 2 cytokines orchestrate tissue repair and fibrosis both directly and indirectly by targeting a wide array of immune and non-immune cell types, including macrophages, fibroblasts, epithelial cells, and endothelial cells (Gieseck et al., 2018). These cytokines have dual roles, as they can facilitate tissue repair by activating tissue progenitor cell populations while also potentially driving pathological fibrosis when dysregulated (Gieseck et al., 2018).

#### 4.1.3 TGF-β signaling in cancer

Transforming growth factor-β (TGF-β) emerges as a central player at the intersection of fibrosis and immune suppression in cancer. It is a potent and pleiotropic cytokine with complex, often contradictory roles in tumorigenesis (Metropulos et al., 2022). While TGF-β effects on tumor cells vary by context, its role in immune evasion appears somewhat consistent across tumor types.

TGF-β functions as a central mediator of immune tolerance, with well-documented immunosuppressive effects (Metropulos et al., 2022). It influences various aspects of the TME, promoting fibrosis while simultaneously suppressing anti-tumor immune responses. This dual action makes TGF-β a particularly important factor in cancer progression and treatment resistance.

TGFβ can promote angiogenesis by upregulating connective tissue growth factor (CTGF), vascular endothelial growth factor (VEGF), and MMP2 (Ruiz-Ortega et al., 2007; Goumans et al., 2009).

Tumor-associated fibrosis represents a significant barrier to effective anti-tumor immunity and successful immunotherapy. Through multiple mechanisms, including the creation of physical barriers, induction of hypoxia, and activation of immunosuppressive signaling pathways, fibrosis creates an environment conducive to tumor growth and immune evasion.

TGF-β emerges as a central mediator of both fibrosis and immune suppression in cancer, making it an attractive therapeutic target. Preclinical studies of combined TGF-β inhibition and immune checkpoint blockade show promise, but translational challenges remain. Novel approaches, including nanoplatform-based delivery systems and combination strategies with chemotherapy, may help overcome these challenges.

Future research should focus on better characterizing the distinct mechanisms through which fibrosis suppresses immune function in different tumor types, identifying biomarkers to predict response to anti-fibrotic therapies, and developing more targeted approaches to modulate the fibrotic microenvironment without compromising beneficial tissue repair functions.

Understanding and targeting tumor-associated fibrosis represents an important frontier in cancer immunotherapy, with the potential to significantly improve outcomes for patients with fibrotic tumors that currently respond poorly to available immunotherapeutic approaches.

#### 4.1.4 Immunosuppressive macrophages in fibrosis

Tumor-associated macrophages (TAMs) play a crucial role in coordinating immune evasion in fibrotic tumors within the TME (Perricone and Lyssiotis, 2024). These cells can both respond to and contribute to the fibrotic environment, often adopting immunosuppressive phenotypes in fibrotic regions. The relationship between macrophages and fibrosis represents a significant mechanism through which tumors escape immune surveillance.

Multiple studies have demonstrated that macrophages are a critical source of TGF-β1 and PDGF in fibrosis, including in the TME (Wynn and Barron, 2010; Li et al., 2024b). These macrophages contribute to creating a dense, fibrotic stroma by secreting pro-fibrotic factors such as TGF-β and PDGF (platelet-derived growth factor), which stimulate fibroblasts to produce ECM components like collagen. TGF-β is identified as the main effector molecule promoting fibrosis by activating fibroblasts to proliferate and produce collagen and other ECM components (Li et al., 2024b).

This fibrotic environment physically and immunologically supports tumor growth, invasion, metastasis, and therapy resistance (Buechler et al., 2021). This collective evidence from experimental models, mechanistic studies, and clinical reviews robustly demonstrates the macrophage-fibroblast axis in generating a fibrotic TME that promotes tumor growth, invasion, immune evasion, and therapy resistance.

For example, in pancreatic ductal adenocarcinoma (PDAC), a highly lethal cancer characterized by a dense fibrotic stroma, macrophages undergo metabolic reprogramming involving collagen scavenging that enhances their profibrotic and immunosuppressive activity. This results in a microenvironment that impedes immune cell infiltration and cytotoxic function, contributing to the cancer’s aggressiveness and resistance to treatments (Ahmad et al., 2021; LaRue et al., 2022; Lorestani et al., 2024).

Furthermore, the potential of cancer-associated fibroblasts (CAFs), specifically FAP+ CAFs, in pancreatic cancer includes both tumor-promoting and tumor-restraining roles. Recent research identified heterogeneity among CAFs in pancreatic ductal adenocarcinoma (PDAC), highlighting a novel interferon-response CAF (ifCAF) subtype with tumor-restraining properties. This ifCAF subtype can be induced by stimulating type I interferon signaling, such as via STING agonists, to suppress tumor cell invasiveness and promote an antitumor immune response through neutrophil modulation.

FAP+ CAFs are highly heterogeneous, with some subsets contributing to tumor growth through extracellular matrix deposition, metabolic support, and immunosuppression, while others (like ifCAFs) can inhibit tumor progression. Understanding and manipulating this diversity could lead to new therapeutic strategies by harnessing tumor-suppressive CAF functions in pancreatic cancer (Cumming et al., 2025).

Regarding fascia treatment in pancreatic cancer, fascia and its connective tissue components significantly influence tumor microenvironments. Fascial remodeling affects cancer behavior, potentially impacting metastasis pathways and immunomodulation. Treatments targeting fascia and connective tissue mechanics, such as myofascial release and osteopathic manipulation, may improve quality of life and symptom management in cancer survivors. Moreover, recognizing fascia’s role could enhance surgical approaches and integrative therapies in oncology (Langevin et al., 2016).

In summary, targeting the diverse CAF populations, especially the tumor-restraining interferon-response CAF subtype, alongside fascia-focused treatments, may offer novel avenues for pancreatic cancer therapy and supportive care. This integration of cellular and tissue-level perspectives advances precision and holistic cancer management (Cumming et al., 2025).

In oral squamous cell carcinoma derived from oral submucous fibrosis, single-cell RNA sequencing and spatial transcriptomics studies have demonstrated an increase in immunosuppressive INHBA-expressing macrophages and proinflammatory cancer-associated fibroblasts (CAFs). These INHBA+ macrophages show the strongest immunosuppressive functions, including high expression of immune checkpoint molecules and suppression of cytotoxic T cells, correlating with poor prognosis and potentially lower sensitivity to immunotherapy (Zhao et al., 2025).

In non-small cell lung cancer (NSCLC), fibrosis driven by specific CAFs promotes recruitment of immunosuppressive macrophages and regulatory T cells, while reducing dendritic and CD8+ T cell infiltration, thereby enhancing T cell exhaustion and weakening anti-tumor immunity despite immune checkpoint therapy interventions (Ghebremedhin et al., 2024; Yang et al., 2025).

The interplay between macrophages and fibrosis is also observed in liver cancer, where pro-fibrotic macrophages contribute to tumor development and immune escape. Chronic infections like hepatitis B virus (HBV) infection induce immunosuppressive macrophage subsets that foster fibrosis and hepatocellular carcinoma progression (Li et al., 2019).

Therapeutically, targeting these immunosuppressive macrophages is a promising strategy. Approaches involve depleting TAMs, reprogramming their phenotype to enhance anti-tumor immunity, inhibiting key signaling pathways such as PI3Kγ, or blocking macrophage receptors like CD206 to modulate macrophage function and reduce fibrosis. Such strategies have shown potential to overcome immune evasion and improve immunotherapy efficacy (Lorestani et al., 2024).

In summary, strong evidence across various cancers supports that immunosuppressive macrophages are central drivers of fibrosis in fascia and other stromal tissues. By remodeling the ECM and suppressing effective immune responses, they create a tumor-permissive microenvironment that promotes tumor progression and resistance to therapy. Targeting these macrophage populations within the fibrotic tumor stroma holds significant therapeutic potential to improve outcomes in cancer patients.

#### 4.1.5 Mechanisms of immune evasion promoted by fibrosis

The balance between collagen degradation and synthesis determines tissue structure and function, while the dysregulation can result in fibrosis (collagen overexpression) or tissue weakening (insufficient collagen). This dynamic remodeling of collagen is a hallmark of many chronic diseases and is central to both tissue repair and the development of pathological scarring. The difference between ECM and stroma lies in their composition and roles within tissue, especially in the context of tumors.

The ECM is an acellular, structural network composed of proteins like collagen, fibronectin, elastin, laminin, proteoglycans, and hyaluronic acid. It forms structures such as the basement membrane and interstitial matrix, providing mechanical support, maintaining tissue homeostasis, and regulating cell behavior. ECM is essentially the non-cellular component secreted by cells, particularly fibroblasts, and is responsible for tissue architecture and physical properties.

The stroma refers to the connective tissue framework of an organ, made up of both the ECM and the specialized connective tissue cells that produce and remodel this matrix. These stromal cells include fibroblasts (including cancer-associated fibroblasts or CAFs in tumors), mesenchymal stromal cells, endothelial cells, adipocytes, and immune cells. The stroma supports parenchymal cells and participates in complex signaling and remodeling processes.

The stiffening of the stromal tissue acts as a powerful mechanical cue that triggers tumor cells to undergo a mesenchymal transition. This process enhances cell motility, invasiveness, and metastatic potential. Additionally, it has been demonstrated to facilitate tumor cell migration, a process known as an EMT (Horta et al., 2023). ECM stiffening orchestrates a tumor-promoting microenvironment by driving abnormal angiogenesis that fails to deliver adequate oxygen, directly contributing to fostering hypoxia through vascular dysfunction, and undermining anti-tumor immunity through both physical and biochemical mechanisms (Mongiat et al., 2016; Bigos et al., 2024; Jiang et al., 2022). These effects work synergistically to promote tumor progression and treatment resistance, making ECM stiffness an attractive therapeutic target for cancer treatment.

As expected, the degree of tissue fibrosis and the level of stromal stiffness have been found to be correlated with the degree of tumor aggression and the patient’s prognosis. The relationship between tumors and fibrosis is dynamic, with fibrosis influencing tumor behavior and vice versa (Piersma et al., 2020; Chandler et al., 2019). Tumor cells secrete inflammatory and pro-fibrotic factors (e.g., TGF-β, IL-1, TNF, IL-6), which activate fibroblasts and myofibroblasts, leading to ECM deposition and tissue stiffening (Chandler et al., 2019). The fibrotic response in tumors, often called desmoplasia, is a defining feature of the TME (Chen et al., 2024).

Fibrosis affects tumors in several keyways. The stiffened, collagen-rich ECM provides structural support and activates signaling pathways that enhance tumor cell proliferation and survival (Chandler et al., 2019). Dense fibrotic tissue physically obstructs the infiltration of immune cells into tumors, thereby creating an immunosuppressive environment that impedes the efficacy of anti-tumor immune responses (Perricone and Lyssiotis, 2024; Quintela-Fandino et al., 2024; Naik and Leask, 2023). An alteration in the ECM and activated fibroblasts are associated with an increase of tumor cell migration and invasiveness (Chandler et al., 2019). As fibrosis impedes drug delivery and contributes to chemoresistance and resistance to immunotherapy by creating a protective niche for tumor cells, it is imperative to consider this factor in prospective treatment strategies (Chandler et al., 2019; Quintela-Fandino et al., 2024).

As indicated by Naik and Leask (2023), elevated levels of tumor fibrosis have been demonstrated to be associated with worth prognosis in a variety of cancers, including HER2-negative breast cancer (Naik and Leask, 2023). The dual role of fibrosis is a subject of considerable interest in the medical community. Most evidence indicate that fibrosis promotes tumor progression, immune evasion, and treatment resistance (Chandler et al., 2019; Perricone and Lyssiotis, 2024; Quintela-Fandino et al., 2024). In particular, the restraining effects of the tumor are as follows: In certain contexts, normal fibroblasts and mesenchymal stem cells have been observed to impede the initiation and growth of tumors. However, as cancer progresses, these cells are often reprogrammed by tumor signals to become cancer-associated fibroblasts (CAFs) that support malignancy (Dorjkhorloo et al., 2024).

**Table 2** gives an overview of the effects of fibrosis on tumors as well as related mechanisms. 

Fibrosis is a central and multifaceted player in cancer biology and remains a major mortality driver, although a direct link between fascial pathology and cancer lacks robust evidence (Liu et al., 2024b). While in rare contexts it can restrain early tumor development, in established cancers fibrosis overwhelmingly facilitates tumor progression, immune evasion, and therapy resistance. Targeting the fibrotic microenvironment is a promising avenue for improving cancer treatment (Piersma et al., 2020; Dorjkhorloo et al., 2024; Chandler et al., 2019).

Tumor-associated fibrosis is a hallmark of many solid tumors and is characterized by the excessive deposition of ECM components, the presence of cancer-associated fibroblasts (CAFs), and increased tissue stiffness. It represents a significant barrier to effective anti-tumor immunity and successful immunotherapy (Jiang et al., 2017). Through multiple mechanisms, including the creation of physical barriers, induction of hypoxia, and activation of immunosuppressive signaling pathways, fibrosis creates an environment conducive to tumor growth and immune evasion. TGF-β emerges as a central mediator of both fibrosis and immune suppression in cancer, making it an attractive therapeutic target. Preclinical studies of combined TGF-β inhibition and immune checkpoint blockade show promise, but translational challenges remain. Novel approaches, including nanoplatform-based delivery systems and combination strategies with chemotherapy, may help overcome these challenges (Metropulos et al., 2022).

Future research should focus on better characterizing the distinct mechanisms through which fibrosis suppresses immune function in different tumor types, identifying biomarkers to predict response to anti-fibrotic therapies, and developing more targeted approaches to modulate the fibrotic microenvironment without compromising beneficial tissue repair functions.

Understanding and targeting tumor-associated fibrosis represents an important frontier in cancer immunotherapy, with the potential to significantly improve outcomes for patients with fibrotic tumors that currently respond poorly to available immunotherapeutic approaches.

### 4.2 Immune cell–ECM interactions

Immune cell subtypes play profound and dynamic roles in the modulation of fibrosis and cancer progression, acting through distinct mechanisms and interactions with the tissue microenvironment (**Table 3**).

**Table 3:** Molecular mechanisms of fibrosis.

Immune cell	Key molecules/pathways	Effect on fibrosis
Macrophages	TGFβ, PDGF, IL-6, MMPs	Promote ECM deposition, fibroblast activation (Greenman and Weston, 2025)
Neutrophils	Proteases, ROS	Modulate ECM, early inflammation (Greenman and Weston, 2025)
NK cells	IFN-γ	Inhibit fibroblast activation, anti-fibrotic (Wick et al., 2010)
Th1 T cells	IFN-γ, IL-12	Suppress fibrosis (Wick et al., 2010; Zhang and Zhang, 2020)
Th2 T cells	IL-4, IL-13	Promote fibrosis (Wick et al., 2010; Zhang and Zhang, 2020)
Th17 T cells	IL-17	Context-dependent, can promote fibrosis (Wick et al., 2010)
B cells	Autoantibodies, cytokines	Modulate immune response, promote fibrosis in autoimmunity (Wick et al., 2010)

#### 4.2.1 Modulation of fibrosis by immune cell subtypes

Macrophages: These are pivotal modulators of fibrosis, with M2 macrophages driving tissue remodeling and anti-inflammatory effects. High M2 macrophage levels characterize the “pro-remodeling” immune subtype and serve as a key feature for fibrotic progression. In contrast, monocytes show a positive correlation with fibrosis and are linked to chronic inflammatory responses.

T Cells: Resting CD4+ T cells are central in the “pro-inflammatory” immune subtype, which exhibits heightened immune activation. CD4+ T cell levels closely interact with monocytes, M0 macrophages, and memory B cells, emphasizing regulatory networks in inflammation-driven fibrosis.

Neutrophils: Their proportion tends to be negatively correlated with fibrosis, acting as potential anti-fibrotic components.

Subtype-specific mechanisms: Patient stratification based on immune cell fractions identifies two main fibrotic mechanisms: chronic inflammation (pro-inflammatory subtype) and remodeling/angiogenesis (pro-remodeling subtype). Distinct gene sets and signaling pathways such as JAK-STAT, mTOR, IL-6 receptor binding, and processes like EMT (epithelial–mesenchymal transition) are linked to these subtypes. **Table 4** gives an overview on the effects and clinical impact of tissue stiffness on tumors. The next paragraphs describe the different effects and pathways.

**Table 4:** Effects and clinical impact of tissue stiffness on tumors.

Effect	Mechanism/pathway involved	Clinical impact	Evidence/source
Proliferation and invasion	FAK, PI3K/Akt, Ras–MAPK, YAP/TAZ	Tumor growth, metastasis	(Takeda et al., 2022; Ajongbolo and Langhans, 2025; Kim and Nam, 2025)
Epithelial–mesenchymal transition (EMT)	Mechanotransduction, microRNAs	Metastasis	(Zhang and Ma, 2012; Mlcochova et al., 2016; Bala et al., 2025)
Therapy resistance	Integrin, CAM-DR, CAM-RR	Treatment failure	(Damiano et al., 1999; Eke and Cordes, 2015)
Immune evasion	Physical barrier, reduced T-cell entry	Poor immunotherapy response	(Anderson et al., 2017; Wang et al., 2025a; Tufail et al., 2025)
Angiogenesis and hypoxia	YAP/TAZ, interstitial pressure	Resistance, poor prognosis	(Choi and Kim, 2024; Sivaraj et al., 2020)
Genomic instability	Nuclear envelope rupture, DNA damage	Increased aggressiveness	(Lim et al., 2016)

#### 4.2.2 Proliferation and invasion

- The pathways FAK, PI3K/Akt, Ras–MAPK, and YAP/TAZ are involved in tumor growth and metastasis. Specifically, hyperactivation of YAP/TAZ promotes tumor cell proliferation and invasion, as shown in colorectal cancer models where targeting YAP decreased invasion via the PI3K/Akt pathway.

#### 4.2.3 Epithelial–mesenchymal transition

- EMT is regulated by mechanotransduction and microRNAs, which influence metastasis. Studies identify multiple miRNAs (e.g., miR-200 family) that regulate EMT processes and thus cancer metastasis, particularly in lung and renal cancers.

#### 4.2.4 Therapy resistance

- Integrins mediate cell adhesion-mediated drug resistance (CAM-DR) and cell adhesion-mediated radioresistance (CAM-RR) by promoting survival signaling and inhibiting apoptosis. This leads to treatment failure in cancers like myeloma by integrin-fibronectin interactions that protect tumor cells from drugs.

#### 4.2.5 Immune evasion

- Tumors create physical barriers like collagen-rich extracellular matrices that restrict T cell infiltration, resulting in poor immunotherapy response. Collagens such as COL3A1 and COL6A1 form barriers preventing T cell entry and attack.

#### 4.2.6 Angiogenesis and hypoxia

- YAP/TAZ signaling is activated under hypoxia, promoting angiogenesis and tumor survival, leading to therapy resistance and poor prognosis. Hypoxia-induced YAP activation involves interaction with HIF-1α and inhibition of the Hippo pathway.

#### 4.2.7 Genomic instability

- Nuclear envelope rupture causes DNA damage and genomic instability, contributing to cancer aggressiveness. This transient or permanent rupture exposes DNA to cytoplasmic damage, promoting mutation and tumor progression.

### 4.3 Impact on cancer progression

Tumor microenvironment: Tumor-associated fibrosis produces extracellular matrix (ECM) deposition and creates hypoxic regions, leading to immune suppression. This environment recruits myeloid-derived suppressor cells (MDSCs) and tumor-associated macrophages (TAMs), altering T cell responses and impeding immune surveillance.

Immune cell clusters and prognosis: Favorable cancer prognosis correlates with clusters that are rich in memory B cells, plasma cells, CD8+ T cells, and activated NK cells. Suppressive subtypes include regulatory T cells and M2 macrophages, which tend to support tumor progression.

Causal connections in cancer subtypes: Comprehensive studies in lung cancer show a differential expression of immune cell subtypes; some exhibit protective effects, while others support tumor growth. Notably, variation exists among histological subtypes such as adenocarcinoma and squamous carcinoma.

Therapeutic implications: Fibrosis impairs immune surveillance and reduces responsiveness to checkpoint blockade immunotherapies. Targeting fibrosis (e.g., through transforming growth factor–β receptor signaling inhibitors) can enhance the efficacy of immunotherapies when combined with chemotherapy.

Immune cell subtypes orchestrate complex signaling and functional networks that shape tissue remodeling, fibrosis development, and cancer progression. Stratifying patients based on immune cell fractions provides a promising path toward precision anti-fibrotic and anti-cancer therapies tailored to immune profiles.

## 5 Therapeutic approaches targeting fascia, ECM, and mechanotransduction

### 5.1 Therapeutic implications of fascial treatment

Myofascial release and other fascial therapies have been investigated as supportive cancer care to reduce pain, inflammation, and tissue stiffness and improve mobility for cancer patients. These therapies may modulate the immune environment and improve the quality of life (Kim et al., 2023b). There is no strong evidence that fascial therapies promote cancer spread; rather, they may provide benefits in palliative and integrative oncology settings. Research in mechanotherapy targeting fascia stiffness and CAFs is ongoing, hinting at future integrative interventions that might complement conventional cancer treatments.

### 5.2 Research needs and emerging findings

Although the fascia–cancer connection is a relatively new field, studies using advanced imaging and molecular profiling are beginning to elucidate the complex biophysical and immunological roles of fascia in cancer progression (Cumming et al., 2025). New discoveries of fibroblast subtypes in connective tissue around tumors reveal that some fascial cells might suppress tumor growth, providing new therapeutic avenues (Cumming et al., 2025). Fascia-informed integrated therapies such as yoga, acupuncture, and massage may contribute to decreasing fascial stiffness and inflammation, potentially impacting tumor dynamics and symptom management.

Evidence supports fascia as a critical component of cancer microenvironments, influencing tumor biology and therapeutic responses. Fascial treatments show promise primarily as adjuncts improving patient symptoms and possibly modulating the tumor environment, with ongoing research exploring direct anticancer potentials (Cumming et al., 2025).

### 5.3 Mechanical and physical therapies

#### 5.3.1 The impact of exercise on the growth and progression of cancer

Epidemiological studies indicate that physical activity counteracts the development of many diseases. Exercise and physical activity have local and systemic effects on the molecular level (Chang et al., 2012). The extent to which these effects are clinically relevant and which tumors are most affected by which effect is still unclear. The mechanistic investigation of the central molecular regulation by targeted training stimuli offers the possibility to improve the overall understanding of tumor development, tumor recognition and tumor control. So far, it has been shown that exercise, depending on stress normatives (type, duration, intensity, frequency), has an effect on numerous biological systems and factors that ultimately cause anticarcinogenic effects (Chang et al., 2012). It is conceivable that bodywork could dislodge tumors and thereby encourage their migration (Langevin et al., 2016). Physical activity promotes vascular normalization in tumors by increasing blood vessel maturity and pericyte coverage, improving oxygen delivery and radiotherapy effectiveness. This reduces hypoxia, a key driver of tumor aggressiveness (Feng et al., 2024b). In colorectal and breast cancer models, combining exercise with radiotherapy reduced tumor growth and metastasis by up to 50% (Tobias et al., 2023; Feng et al., 2024b).

Exercise may also enhance the efficacy of cancer treatments and improve outcomes in pancreatic cancer patients through several mechanisms: Exercise can impact tumor growth through an interplay of muscle-derived mediators (myokines), alterations of tumor vascularization and metabolism, and changes in anti-tumor immunity (Brummer et al., 2023). Thereby, exercise-mediated adaptations on the host and tumor sites can influence each other (double-headed arrow) (Brummer et al., 2023). Emerging evidence indicates that exercise can increase the immune response against pancreatic tumors (Kurz et al., 2022), increase sensitivity to cancer drugs (Kurz et al., 2022), mitigate treatment side effects and improve the quality of life (Luo et al., 2021), and potentially inhibit tumor growth directly (Hsueh et al., 2022). However, more rigorous clinical trials are needed to confirm these findings and determine optimal exercise prescriptions for pancreatic cancer patients (Kurz et al., 2022; Luo et al., 2021; O'Connor et al., 2021). Myofascial release and stretching exercises have an impact on the stiffness of the ECM and the TME. Increased matrix rigidity associated with the fibrotic reaction is documented to stimulate intracellular signaling pathways that promote cancer cell survival and tumor growth (Kodama et al., 2023).

#### 5.3.2 Overview

Regular physical activity has a significant influence on cancer risk, growth, and progression. Research shows that exercise not only reduces the likelihood of developing certain cancers but also slows tumor growth, improves treatment outcomes, and enhances quality of life for cancer patients (Wang and Zhou, 2021; Feng et al., 2024b; Betof et al., 2013). The inter-relationship between inflammation, cancer, and innate immunity has recently gained acceptance; however, the underlying cellular and molecular mechanisms behind this relationship are yet to be solved. Several studies suggest that physical-exercise-mediated induction of immune cells elicit anti-tumorigenic effects. This indicates the potential of exercising in modulating the behavior of immune cells to inhibit tumor progression. However, further mechanistic details behind physical-exercise-driven immunomodulation and anticancer effects have to be determined.

For example, exercise stimulates the mobilization and infiltration of immune cells, such as natural killer (NK) cells and cytotoxic T cells, into the TME. These immune cells attack and suppress cancer cells, slowing tumor growth and potentially reducing metastasis (Wang and Zhou, 2021; Rundqvist et al., 2020; Spanoudaki et al., 2023).

#### 5.3.3 Mechanisms: How exercise affects cancer

##### 5.3.3.1 Immune system modulation

Exercise-induced cytokines (e.g., IL-6) help mobilize additional immune responses and reduce inflammation, which is linked to cancer progression (Feng et al., 2024b, 2024). Regular physical activity upregulates cancer-fighting immune cells, particularly cytotoxic T cells. Exercise alters T cell metabolism, which enhances their anti-cancer activity through the release of metabolites (such as lactate) that boost T cell function and stimulate apoptosis in cancer cells. Skeletal muscle contraction during exercise leads to the secretion of myokines, like IL-6 (Rundqvist et al., 2020) and secreted protein acidic and rich in cysteine (SPARC), which reduce tumor-promoting inflammation and help induce apoptosis in certain cancers, contributing to a lower risk of recurrence, even if it is just a single bout (Bettariga et al., 2025).

SPARC is an exercise-induced myokine primarily secreted by skeletal muscles but also expressed in bone and adipose tissues. It has diverse biological roles including ECM remodeling, osteogenesis, angiogenesis, wound healing, and modulation of inflammation and fibrosis. SPARC represents a promising therapeutic target for treating conditions such as osteopenia, sarcopenia, type 2 diabetes, chronic inflammation, and fibrosis (Piccirillo, 2019; Aoi et al., 2013; Mathes et al., 2022).

##### 5.3.3.2 Metabolic and hormonal effects

Physical activity lowers circulating levels of glucose and insulin-like growth factors, which are known to fuel cancer cell proliferation. Exercise activates anti-cancer pathways (e.g., AMPK) and suppresses pro-cancer signaling (e.g., PI3K/Akt/mTOR) (Feng et al., 2024a, 2024). Myokines released from muscles during exercise (such as oncostatin M, irisin, and SPARC) directly inhibit cancer cell growth and promote apoptosis (programmed cell death) (Spanoudaki et al., 2023).

##### 5.3.3.3 Exercise-induced extracellular vesicles and systemic communication

Physical activity also leads to changes in circulating extracellular vesicles (EVs), which carry enzymes, metabolites, and regulatory factors that influence tumor bioenergetics and immune activity. These vesicles are integral for intercellular communication and may contribute to systemic anti-cancer effects by modifying OXPHOS and glycolytic fluxes (Puurand et al., 2024).

##### 5.3.3.4 Mechanotransduction and physical force effects

Physical forces (mechanical stress, substrate rigidity, fluid shear, tension) in the TME are critical in regulating cancer progression and metastasis by stimulating mechanotransduction pathways. These cues affect the cytoskeleton and cell–ECM adhesion, influencing cell division, survival, migration, and ECM remodeling. Key molecular systems involved include integrins, focal adhesions (FAK), and cytoskeletal proteins, which drive signaling pathways such as ERK, Rho-GTPase, and contractility responses (Kim et al., 2023a; Agrawal et al., 2025; Kumari et al., 2023).

This **Table 5** highlights how physical forces at the cellular and tissue levels influence cancer progression and patient outcomes through mechanotransduction, immune interactions, and systemic fitness effects (Bettariga et al., 2025; Li et al., 2024a; Peng et al., 2024; Angeli et al., 2025; Agrawal et al., 2025).

**Table 5:** Physical force and cancer.

Physical force aspect	Effect on cancer	Mechanism or observation	Evidence/source
Mechanical forces in tumor microenvironment (TME)	Promote tumor progression, metastasis, therapy resistance	Mechanical deformation activates mechanosensitive signaling pathways altering proliferation, migration, invasion, survival	(Angeli et al., 2025)
Compressive stress on tumor cells	Alters metabolism, inhibits proliferation in spheroids, promotes invasiveness	Physical stress affects cell behavior and ECM interaction	(Liu et al., 2020)
Force exerted by immune cells (T cells) on cancer ligands	Regulates PD-1 checkpoint activation, influences immune response efficacy	Physical force necessary for PD-1 ligand function, enabling immune cell killing of cancer	(Li et al., 2024a)
Mechanical force-mediated intercellular interactions (e.g., fibroblasts and cancer cells)	Enhance proliferation, invasion, migration, metastasis	Activation of signaling pathways (Rho–ROCK–MLC, FAK-Src-ERK, JNK) by mechanical cues	(Peng et al., 2024)
Muscle strength and physical fitness in cancer patients	Associated with lower risk of death, improved survival	Possibly via systemic health, immune function, and reduced adverse effects of cancer therapies	(Bettariga et al., 2025)
Solid stress and ECM stiffness in tumors	Promotes cancer cell aggressiveness, EMT, and tumor spread	Increased mechanical stress triggers secretion of pro-metastatic cytokines, alters cell mechanics	(Agrawal et al., 2025)
Mechanical signatures in cancer metastasis	Promote irregular solid and fluid stresses contributing to tumor progression and treatment resistance	Alters tumor biomechanics that facilitate metastasis and reduce therapy response	(Agrawal et al., 2025)
Chronic stress effects via mechanical signaling	Alters tumor microenvironment, increases angiogenesis, immunosuppression, and tumor growth	Neuroendocrine–immune interaction modulated by stress hormones impacting tumor	(Tian et al., 2021; Hong et al., 2021)

##### 5.3.3.4 TME and angiogenesis

Exercise can normalize tumor blood vessels, improving oxygen delivery and reducing tumor hypoxia, which makes tumors less aggressive and more responsive to treatments (Feng et al., 2024b, 2024). It also reduces the number of tumor-associated macrophages and neutrophils that support cancer growth and spread (Feng et al., 2024a, 2024).

##### 5.3.3.5 Genetic and epigenetic regulation

Exercise alters the expression of microRNAs (miRNAs) involved in cancer pathways, upregulating tumor suppressors and downregulating oncogenes (Papadopetraki et al., 2022). Changes in gene expression can reduce tumor proliferation, invasion, and angiogenesis.

##### 5.3.3.6 Preclinical and clinical studies of exercise in cancer care

Animal studies: Mice with cancer that exercised regularly showed slower tumor growth and improved survival compared to inactive controls. The anti-cancer effects were linked to the increased activity of cytotoxic T cells (Rundqvist et al., 2020).

Human studies: Epidemiological data indicate that physically active individuals have a reduced risk of cancer progression and mortality. Risk reductions range from 15% to 67% for cancer-specific and all-cause mortality (Betof et al., 2013; Mabena et al., 2025).

Clinical observations: Exercise before and after a cancer diagnosis is associated with lower risks of disease progression and death (Mabena et al., 2025).

In summary, exercise emerges as a potent modulator of the tumor microenvironment (TME), with robust evidence from both *in vivo* and *in vitro* studies substantiating its inhibitory effects on cancer progression. The role of myokines as critical mediators in this context underscores their potential as key targets for advancing mechanistic understanding of how exercise exerts its antineoplastic effects (Gunasekara et al., 2024).

#### 5.3.4 Exercise as part of cancer care

Exercise is recommended as an adjunct to standard cancer treatments due to its ability to:

- Reduce treatment-related side effects (e.g., fatigue, cachexia)
- Improve physical function and quality of life
- Enhance the efficacy of anti-cancer therapies by modifying the TME (Feng et al., 2024b)

##### 5.3.4.1 Complementary movement (body work) therapies: yoga and stretching

Emerging preclinical and clinical evidence support integrating yoga and structured stretching into oncology care to alleviate treatment-related symptoms, enhance functional capacity and the quality of life, and potentially modulate mechanobiological pathways that influence tumor microenvironments (Langevin et al., 2016).

Tumors generate elevated solid stress pressures exceeding normal tissue by four- to 10-fold, with breast cancer tissue stiffness rising from ~0.2 kPa in healthy states to >4 kPa in malignancy (Agrawal et al., 2025; Berrueta et al., 2018a). This pathological mechanotransduction promotes oncogenic behaviors, including enhanced cell proliferation, matrix metalloproteinase-driven invasion, growth-factor-mediated angiogenesis, and immune suppression via physical barriers impeding T-cell infiltration. Stretching interventions normalize tissue mechanics by reducing accumulated solid stress, facilitating extracellular matrix (ECM) remodeling, and disrupting pro-tumorigenic feedback loops (Wang et al., 2019).

In Berrueta et al. (2018a), female FVB mice (*n* = 66) bearing orthotopic p53/PTEN double-null mammary tumors underwent daily 10-min stretching of the affected tissue for 4 weeks versus no-stretch controls (Berrueta et al., 2018a). Tumor volume at endpoint was 52% smaller in the stretch group (*p* < 0.001), accompanied by activated cytotoxic immune responses, elevated specialized pro-resolving mediators, and reduced stromal fibrosis, without systemic interventions. Complementing this, He et al. (2025) developed the first voluntary stretching model using a “high cage” system (elevated food/water in Noldus PhenoTyper cages), confirming increased vertical elongation behaviors via 24/7 video tracking. In MET-1 tumor-bearing FVB mice, high-cage stretching slowed tumor growth comparably to voluntary wheel running, via distinct plasma proteomic profiles (e.g., adiponectin upregulation) independent of locomotion, body weight, or composition changes (He et al., 2025).

Gentle daily 10-min stretching reduces local connective tissue inflammation and fibrosis (Berrueta et al., 2018b). Berrueta et al. (2023) detailed this in male C57BL/6 mice with subcutaneous carrageenan-induced lesions, randomized to twice-daily 10-min stretching or controls. Assessments (ultrasound, LC-MS/MS lipidomics, flow cytometry, single-cell RNA-seq, immunohistochemistry) revealed significantly smaller lesion sizes at 96 h (pro-chronic transition), with boosted 17-HDHA (pro-resolving intermediate) at 48 h, reduced pro-inflammatory (PGE2, PGD2) and select pro-resolving mediators by 96 h, sustained neutrophil activity, and upregulated dual macrophage genes (Nos2 pro-inflammatory; Arg1 pro-resolving). Spatial analysis showed Arg1 localized to lesion rims, suggesting that mechanical forces enhance resolution via balanced immune crosstalk and tissue repair without suppressing acute inflammation (Berrueta et al., 2023).

Yoga and adapted stretching routines prove safe for most patients during/after treatment, with serious adverse events exceedingly rare when tailored (e.g., avoiding acute post-surgical phases) (Saraswathi et al., 2021; Freguia et al., 2024; Cramer et al., 2013). Consistent practice (three to five sessions/week, totaling 150–220 min) yields clinically meaningful reductions in cancer-related fatigue, anxiety, depression, sleep disturbances, systemic inflammation (↓IL-6, TNF-α), cortisol levels, and autonomic imbalance, alongside improved immune surveillance (Zetzl et al., 2021; Kiecolt-Glaser et al., 2014; Liu et al., 2024a). Stretch-focused protocols further promote lymphatic drainage, alleviate musculoskeletal pain, and mitigate lymphedema (especially with progressive strengthening) in breast cancer cohorts; early integration during chemotherapy/radiotherapy blunts fatigue and preserves sexual/urinary function in prostate/breast cancer patients (Freguia et al., 2024; Wei et al., 2019).

Robust evidence from randomized controlled trials and meta-analyses affirms physical therapies (yoga, stretching) for supportive endpoints—fatigue, pain, anxiety, lymphedema, quality of life—across active treatment and survivorship phases (Wang and Zhou, 2021). Epidemiologic data link physical activity to 15%–67% reductions in cancer-specific/all-cause mortality, independent of direct tumor effects (Betof et al., 2013; Mabena et al., 2025). Preclinical findings extend to broader exercise benefits like tumor vascular normalization, reduced tumor-associated macrophages, and enhanced cytotoxic T-cell infiltration (Feng et al., 2024b; Rundqvist et al., 2020). However, no human RCTs demonstrate fascial-targeted stretching yields tumor regression, progression delay, or survival gains; the largest trials (e.g., CHALLENGE, INTERVAL) attribute the benefits to multifactorial (immune, metabolic, hormonal) pathways. Thus, mechanical interventions should be framed as established supportive care with plausible, preclinical-supported tumor-modulating potential, meriting dedicated RCTs for validation within integrative oncology paradigms (Langevin et al., 2016).

### 5.4 The relationship between stress axis, immune system, and fascia

The hypothalamic–pituitary–adrenal (HPA) axis is a central regulator of the body’s stress response, linking the nervous, endocrine, immune, and fascial systems. Stress activates the HPA axis, leading to the release of catecholamines and cortisol from the adrenal glands (Barsotti et al., 2021). These hormones, together with sympathetic nervous system activation, stimulate immune cells embedded within fascia (e.g., macrophages, mast cells, neutrophils), which, in turn, release signaling molecules that can reshape the fascia structure.

Chronic or dysregulated activity of the HPA axis is strongly associated with psychiatric disorders such as depression, bipolar disorder, schizophrenia, anxiety, and PTSD, with genetic factors also contributing to variability in individual stress responses.

The vagus nerve further integrates these systems by transmitting signals between fascia and the brain while also serving as a key pathway of the gut–brain axis. Since the gut produces many brain-like neurotransmitters, both fascia and vagal pathways critically shape the overall communication between body and brain (Jin et al., 2024). This shows how the HPA axis, fascia, immune system, and vagus nerve all interconnect within the body’s stress response, with direct implications for both mental and physical health. However, fascia plays a key role in the quality of the whole conversation. .

### 5.5 Mechanobiological and translational strategies

#### 5.5.1 Fascial-directed therapies as adjuncts to systemic treatment: The drug delivery rationale

One of the most physiologically compelling arguments for fascial-directed intervention in oncology is not direct cytotoxicity but the reduction of mechanical barriers to drug and immune-cell delivery within the TME. Solid tumors generate elevated mechanical stress arising from tumor cell proliferation, ECM compaction, and desmoplastic fibrosis that exceeds normal tissue levels by four- to 10-fold ( (Agrawal et al., 2025). This solid stress compresses intratumoral blood and lymphatic vessels, creating hypo-perfusion, hypoxia, and elevated interstitial fluid pressure, all of which impair the penetration of both small-molecule drugs and immunotherapy agents (Papageorgis et al., 2017; Mpekris et al., 2018). Dense peri-tumoral fascial and stromal barriers additionally restrict T-cell trafficking and contribute to the immune-excluded tumor phenotype that predicts poor response to checkpoint inhibition (Yui and Oudin, 2024; Mai et al., 2024).

The most direct experimental evidence for the therapeutic relevance of solid stress reduction comes from work with the anti-fibrotic compound tranilast. Papageorgis et al. (2017) demonstrated in MCF10CA1a and 4T1 breast tumor models that tranilast significantly reduced solid stress, decompressed intratumoral blood vessels, decreased interstitial fluid pressure to near-normal levels, and enhanced the efficacy of both chemotherapy and nano-therapy in a size-independent manner (Papageorgis et al., 2017). A complementary mathematical model by Mpekris et al. (2018) confirmed that stress alleviation with tranilast improves tumor perfusion and drug delivery and that this strategy could be further combined with vascular normalization to maximize therapeutic outcomes (Mpekris et al., 2018).

Translational confirmation of this concept was provided by Panagi et al. (2024) in a repurposed phase II clinical trial evaluating the mast cell stabilizer ketotifen, which inhibits mast-cell-driven CAF proliferation and ECM deposition, in human sarcoma patients. Ketotifen successfully suppressed CAF proliferation and reduced ECM stiffness, measured by shear wave elastography, which was accompanied by increased vascular perfusion, improved tissue oxygenation, and enhanced T-cell infiltration; these changes translated into improved chemo-immunotherapy efficacy and acquisition of tumor antigen-specific immune memory (Panagi et al., 2024). Further preclinical validation was provided in KPC pancreatic tumor models, where ketotifen reduced tumor stiffness (elastic modulus from ~40 to ~20 kPa within 3 days) and significantly enhanced the delivery and efficacy of Doxil and other nanomedicines (Angeli et al., 2026). Recent work in PDAC and melanoma models has also confirmed that ketotifen-mediated stromal modulation improves vascular perfusion and enhances the efficacy of nanomedicine-based combination regimens (Angeli et al., 2026). Collectively, these studies demonstrate a mechanistically coherent pathway: reducing ECM/fascial stiffness → decompressing vessels → normalizing perfusion → improving drug and immune-cell delivery.

For LOX-targeted approaches, inhibition of LOXL2 reduces established fibrosis and promotes tissue remodeling (Zhang et al., 2022); broad-spectrum LOX inhibition with β-aminopropionitrile (BAPN) reduces collagen cross-linking, normalizes ECM architecture, and, in preclinical models, improves immune cell infiltration and drug delivery in breast and pancreatic cancers (Yui and Oudin, 2024; Feng et al., 2024b).

Physical fascial interventions may act through biophysically analogous mechanisms. By reducing peri-tumoral tissue stiffness and inter-fascial tension, myofascial release and controlled stretching could theoretically normalize vascular compression, reduce interstitial fluid pressure gradients, and facilitate T-cell trafficking into immune-excluded tumors. Mechanistically, Berrueta et al. (2018b) demonstrated that daily stretching for 4 weeks reduced tumor volume by 52% (below normal tissue stiffness ~0.2 kPa vs. cancerous tissue >4 kPa), associated with reduced stromal TGF-β, collagen density, inflammatory mediators, activation of cytotoxic immune responses, and elevation of specialized pro-resolving lipid mediators (Berrueta et al., 2018b). These mechanistic changes are directly relevant to the drug delivery hypothesis.

It must be stated plainly, however, that this integration framework is currently based on preclinical evidence, mechanistic inference, and early-phase clinical data. No randomized controlled trial in humans has yet demonstrated that fascial-directed physical therapies improve systemic drug efficacy, tumor perfusion, or survival when combined with chemotherapy or immunotherapy. The clinical translation agenda must therefore prioritize (1) imaging sub-studies using shear-wave elastography and dynamic contrast-enhanced MRI to quantify changes in tumor stiffness and perfusion following physical interventions, (2) pharmacokinetic sub-studies within existing exercise oncology trials to assess intratumoral drug concentrations, and (3) prospective trials explicitly evaluating the combination of fascial mechanotherapy with immune checkpoint inhibitors in tumors with high fibrotic burden, modeled on the ketotifen clinical trial design (Panagi et al., 2024).

**Table 6** highlights a mechanistic continuum from robust *in vitro*/animal data on stiffness-induced EMT (e.g., YAP/TAZ nuclear translocation, TWIST1 release) to clinical observations of fascial infiltration as an independent prognostic factor in sarcomas, emphasizing the fascia’s underappreciated role in the tumor microenvironment. While LOX/LOXL2 inhibition and solid stress reduction yield emerging translational efficacy for improved drug delivery and therapy response, stretching or fascial therapies lack RCT evidence beyond quality-of-life benefits, representing hypothesis-generating opportunities. These findings advocate integrating mechanobiological assays into exercise oncology trials to bridge preclinical insights with physiological outcomes in cancer care.

#### 5.5.2 Modulating tissue stiffness as an intervention

Tissue stiffness refers to the mechanical rigidity of biological tissues, which is determined by cellular components and the organization of the extracellular matrix (ECM). Tissue stiffness can change due to injury, pathology (such as fibrosis or cancer), and normal aging. Modulating this stiffness, either increasing or decreasing it, can have therapeutic implications.

Physical exercise and stretching: Specific exercise modalities such as resistance training, aerobic training, plyometric training, and stretching can induce adaptations in tissue stiffness. Resistance training typically increases tendon stiffness in healthy individuals, while aerobic training and stretching can decrease arterial stiffness. Stretching, especially passive static stretching, is found to reduce muscle–tendon unit (MTU) stiffness, with diverse effects depending on muscle type and the protocol used (Thomas et al., 2022).

Targeting ECM components and remodeling: Modifying collagen, fibronectin, laminin, elastin, and glycosaminoglycans (GAGs) in the ECM can change tissue stiffness. Pharmacological agents that affect ECM composition (such as LOX inhibitors or tranilast, an antiallergic drug) are under investigation for their ability to soften tissues and improve outcomes in cancer immunotherapy by enhancing immune cell infiltration. Cell signaling pathways (e.g., Rho kinase or YAP/TAZ activation) also govern ECM synthesis and hence tissue stiffness (Mai et al., 2024; Zhang and Zhang, 2025).

Materials and scaffold engineering: In tissue engineering, scaffold stiffness can be tuned by cross-linking, incorporating reinforcing biomaterials or modifying the architecture. Hydrogels with adjustable stiffness, such as those containing collagen and alginate, are used to mimic physiological tissue properties and study cellular responses (Han et al., 2024).

Cancer therapy: Modifying ECM stiffness has shown promise in cancer treatment by improving immunotherapy efficacy. Softer matrices can facilitate T-cell migration and infiltration, enhancing immune responses against tumors (Zhang and Zhang, 2025; Mai et al., 2024).

Fibrosis and urological disease: Increased tissue stiffness is a hallmark of fibrosis and certain urological diseases. Mechano-based therapies aim to normalize tissue mechanics by targeting increased stiffness and associated cellular changes, such as via the renin–angiotensin system in cancer (Martinez-Vidal et al., 2021).

Cardiovascular and connective tissue disease: Adjusting cardiac tissue stiffness using dynamic hydrogels has been shown to reverse detrimental fibroblast activation, indicating potential therapies for heart disease.

#### 5.5.3 Mechanisms underlying stiffness modulation

Mechanical cues (substrate rigidity, force transmission, mechanotransduction) guide cell behavior, differentiation, and pathological transformation.

Chemical signals (growth factors, ECM-modifying drugs) influence tissue remodeling and stiffness at the cellular and molecular levels.

Altered cellular responses: Cell shape, adhesion, migration, and fate are all affected by changes in ECM stiffness; thus, intervening at the mechanical level can reshape tissue function and pathology.

Modulating tissue stiffness is both a diagnostic marker and a therapeutic target across diverse fields, especially cancer, fibrosis, cardiovascular, and connective tissue diseases. Interventions include exercise, pharmacological drugs, and engineered biomaterials. Emerging strategies aim to restore or normalize tissue stiffness to improve disease outcomes and regenerative capacity (Mai et al., 2024; Thomas et al., 2022; Zhang and Zhang, 2025; Martinez-Vidal et al., 2021; Han et al., 2024).

This approach offers hope for both predicting disease progression and optimizing interventions personalized to patient-specific tissue mechanics.

#### 5.5.4 Combining mechanotherapy with standard treatments

Combining mechanotherapy with standard treatments is a rapidly advancing strategy in multiple therapeutic areas, especially cancer and physical therapy, because it leverages mechanical modulation alongside pharmaceuticals, immunotherapies, or other conventional modalities to enhance clinical outcomes.

Mechanotherapeutics (e.g., agents that reduce tissue stiffness) can decompress tumor blood vessels, alleviate hypoxia, and promote better drug and oxygen delivery. Examples include drugs like losartan or ketotifen, which alter the stiffness and stress within the tumor microenvironment (TME), making tumors more permeable to treatments (Koutsi et al., 2025). Sonopermeation (using ultrasound to increase vessel permeability) further boosts blood flow and enhances the delivery of chemotherapy, immunotherapy, or nanomedicine (Neophytou et al., 2025, Koutsi et al., 2025).

Physical therapy uses mechanotherapies (such as load-based exercise, joint mobilization, shockwave therapy) to stimulate tissue adaptation and healing. When integrated with regenerative therapies (stem cell, scaffold implants), mechanotherapy improves the integration and restorative capacity of the introduced biological materials (Thompson et al., 2016, Ng et al., 2017). Optimizing physical therapy for tissue regeneration demands personalized assessment, exercise prescription, manual techniques, synergy with regenerative therapies, progressive adaptation, and focus on the patient’s functional and lifestyle goals. This approach maximizes the biological and functional healing potential for each individual (Khan and Scott, 2009, Chen et al., 2022, Mureed et al., 2024, Ng et al., 2017).

Combining mechanotherapy with standard treatments has the potential to improve therapeutic efficacy by modulating physical barriers and enhancing tissue responsiveness to pharmacological and regenerative agents. Emerging evidence indicates benefits such as improved drug penetration and augmented tissue repair; however, these effects remain context-dependent and are influenced by parameters such as dosing, sequencing, and timing, which are still being optimized through experimental and computational studies (Neophytou et al., 2025, Thompson et al., 2016). While preliminary findings are encouraging, further controlled investigations are required before this combined approach can be robustly translated into clinical practice.

In summary, integrating mechanotherapy with established treatment modalities leverages mechanobiological principles to enhance therapeutic outcomes; however, its clinical efficacy and mechanistic specificity require further validation before it can be regarded as a new standard for multi-modal interventions in disease management and tissue regeneration.

#### 5.5.5 Future perspectives for targeting the mechanobiological axis in cancer

The mechanobiological axis, encompassing tissue stiffness, ECM remodeling, tumor solid stress, and biophysical signaling, is rapidly becoming a transformative therapeutic target in oncology.

Key outlooks include the following:

- Interdisciplinary innovation, combining biophysical, molecular, and immunological therapies, will drive significant advances.
- Personalized mechanomedicine is likely to become a cornerstone of cancer care, leveraging both mechanical and molecular profiling of patient tumors.
- Clinical translation will depend on further validation, improved measurement technologies, and successful integration of biomechanical diagnostics into routine oncology practice.

This shift toward targeting the mechanobiological axis marks a new era: it promises better outcomes for therapy-resistant and metastatic cancers, greater efficacy of current and next-generation treatments, and sustainable long-term disease control through precise modulation of the tumor microenvironment’s physical properties (Zhuang et al., 2023; Kalli et al., 2023; Chen et al., 2023).

### 5.6 The role of mechanical factors for the TME

Cancer progression and treatment response are significantly influenced by the TME, with fibrosis emerging as a critical factor in immune evasion and therapy resistance. The development of neoplasms is dependent on the intercellular communication between cancer cells and other cells in the TME. The growth and metastasis of cancer cells are influenced by the interactions between immune cells and cancer cells within the TME (Wright et al., 2023). The underlying cause of problems in the ECM is inflammation (Riley and Bradshaw, 2020). The ECM is an inherently dynamic structure, and mounting evidence indicates that ECM proteins establish a physical and biochemical niche for cancer stem cells (CSCs) (Nallanthingal et al., 2019).

Tumors could generate their own infrastructure, which can be defined as a complex network of interconnected vessels, immune cells, signaling molecules, and ECMs (Nallanthingal et al., 2019). ECMs consist of collagen-rich support scaffolding that provides a structural framework for cells to proliferate and differentiate. Thirdly, the mechanical interaction between intracellular and extracellular forces has been demonstrated to promote the invasive potential of tumor cells within tumors. The ECM is a crucial component of the TME, especially in solid cancers. In many solid tumors, the ECM can constitute up to 60% of the total tumor mass (Anderson and Simon, 2020). This extensive presence highlights its significant influence on tumor biology.

Cancer cells respond to extracellular matrix (ECM) stiffness in a complex and dynamic manner. Rather than a simple linear relationship, there is evidence that cancer cells exhibit optimal responses within a specific range of ECM stiffness, which can influence their proliferation, migration, invasion, and other malignant behaviors. Cancer cells respond most aggressively within an optimal range of ECM stiffness that supports their proliferation, migration, and invasion. Both very soft and very stiff matrices can limit these behaviors, and the exact optimal range may vary by cancer type, genetic background, and microenvironmental context (Wang et al., 2024; Zhang and Zhang, 2025; Jahin et al., 2023). This nuanced response underscores the importance of ECM stiffness as a therapeutic target and as a regulator of tumor progression.

The study of Ahmadzadeh et al. (2017) presents a comprehensive mechanochemical model that elucidates how cancer cells invade from primary tumors through a complex two-way feedback mechanism between cellular contractility and extracellular matrix (ECM) properties (Ahmadzadeh et al., 2017). The research reveals novel insights into the nonlinear mechanics governing cancer metastasis and provides both theoretical predictions and experimental validation (Ahmadzadeh et al., 2017).

#### 5.6.1 Core two-way feedback mechanism

The fundamental discovery is a bidirectional feedback loop between cell contractility and matrix fiber realignment. This mechanism operates through two interconnected processes, namely:

Cell-to-matrix effects: Contractile forces exerted by tumor cells realign and induce strain stiffening in the surrounding ECM fibers, particularly in the radial direction perpendicular to the tumor-matrix boundary.

Matrix-to-cell effects: The resulting fiber realignment and stiffening create large tensile forces on cells, activating mechanosensitive pathways including Ca²^+^ and Rho signaling cascades that further enhance cellular contractility.

This creates a self-amplifying cycle where increased contractility leads to greater matrix alignment, which, in turn, promotes even higher contractility levels, ultimately enabling cells to break free from intercellular adhesions and invade the surrounding tissue.

#### 5.6.2 Critical stiffness and biphasic invasion behavior

The model identifies a critical matrix stiffness (approximately 0.15 kPa) that represents a threshold for invasion initiation. Below this critical value, tumor spheroids remain stable because the driving force cannot overcome cell-cell adhesions. Above this threshold, invasion becomes energetically favorable and occurs spontaneously.

Remarkably, the research reveals a biphasic relationship between matrix stiffness and invasion rate. The invasion rate initially increases with matrix stiffness until reaching an optimal value of approximately 0.6 kPa and then decreases at higher stiffnesses due to reduced pore size that restricts cell movement. This explains why intermediate matrix stiffness provides optimal conditions for invasion, with implications for understanding how tumor microenvironments promote metastasis.

#### 5.6.3 Cell polarization and morphological changes

The model successfully predicts that cell elongation correlates positively with matrix fiber alignment. In fibrous matrices, cells develop highly polarized, spindle-like morphologies with aspect ratios increasing proportionally to matrix stiffness and alignment. This polarization results from differential contractility: high contractility in the radial direction (aligned with stiff matrix fibers) and lower contractility in the transverse direction.

Conversely, cells in non-fibrous matrices remain rounded and unpolarized, underscoring the crucial role of matrix microstructure in determining cell morphology and invasive potential.

#### 5.6.4 Experimental validation

The theoretical predictions were rigorously validated using melanoma cell spheroids embedded in collagen matrices of varying concentrations (0.1–2.0 mg/mL):

- Low concentration (0.1 mg/mL): Spheroids remained stable with rounded cell morphology, confirming the existence of a critical threshold.
- Intermediate concentrations (0.5–1.0 mg/mL): Spheroids disintegrated with cells adopting elongated morphologies and actively invading the matrix, validating the critical stiffness prediction.
- High concentration (2.0 mg/mL): Despite cell elongation, invasion was significantly reduced due to restricted pore size, confirming the biphasic behavior.

Additional validation included experiments with Rho pathway inhibitors (Y27632), which reduced invasion in intermediate concentrations, confirming the model’s mechanistic predictions.

#### 5.6.5 Superdiffusive migration kinetics

The model predicts that cancer cell migration follows superdiffusive kinetics rather than simple Brownian motion. The propagation distance increases with time with an exponent greater than 0.5, indicating directed migration under the influence of mechanochemical driving forces rather than random diffusion. This finding provides quantitative insight into how the two-way feedback mechanism creates a persistent, directed cell movement.

#### 5.6.6 Clinical and therapeutic implications

The research has significant translational potential by demonstrating that morphological and structural changes in the tumor microenvironment, such as elevated rigidity and fiber alignment, can serve as prognostic indicators of malignant phenotype. The model explains how these mechanical alterations precede and promote cell invasion, providing potential targets for therapeutic intervention.

The study also reveals that increasing ligand density in the ECM decreases the critical stiffness required for invasion, suggesting that both the mechanical and adhesive properties of the tumor microenvironment contribute to metastatic potential.

#### 5.6.7 Mechanistic insights and novel contributions

Unlike previous computational models that focused primarily on protrusion and traction forces, this research uniquely incorporates the reciprocal relationship between cell contractility and matrix mechanics. The model explains how cells acquire the contractility necessary to overcome intercellular adhesions through mechanosensitive feedback mechanisms, providing a more complete picture of the invasion process.

The findings demonstrate that mechanical principles mediated by cell contractility and ECM nonlinearity play crucial roles in determining invasion phenotype, offering new perspectives on how physical forces drive cancer progression and potentially informing the development of mechanically targeted therapeutic strategies (Ahmadzadeh et al., 2017).

The study presents a computational and experimental model describing a two-way mechanical feedback loop between cancer cell contractility and the extracellular matrix (ECM). This feedback links cell-generated stress with matrix fiber realignment and strain stiffening, forming a self-reinforcing mechanism that promotes invasion.

Key findings include the following:

Critical stiffness threshold: Cells invade the ECM only when matrix stiffness exceeds a certain level, making invasion energetically favorable by weakening cell–cell adhesions.

Biphasic stiffness, invasiveness relationship: Intermediate ECM stiffness maximizes invasion potential; too soft or too stiff matrices reduce invasion.

Cell elongation and fiber alignment: There is a strong positive correlation between matrix fiber alignment and cell polarization. Elongated cells emerge in fibrous, aligned matrices, while cells remain rounded in nonfibrous environments.

Mechanosensitive signaling: Pathways involving Ca²^+^ and Rho signaling enhance myosin activity, increasing contractility and reinforcing the matrix alignment-polarization loop.

Mechanical feedback control: The interplay between cell contractile stress and the nonlinear elasticity of fibrous ECM governs cell shape, motility, and invasive phenotype.

Overall, the model demonstrates that cancer invasion is governed by nonlinear mechanical feedback between cellular contractility and matrix mechanics, providing a predictive framework that links ECM stiffness and structure to malignant cell behavior and morphological transformation.

#### 5.6.8 Underlying mechanisms

Soft ECM:

- Promotes a rounded, less invasive cell phenotype.
- Reduces cell migration due to insufficient traction forces.
- Cells remain clustered, as the force required to break cell–cell adhesions is not met (Geiger et al., 2019; Jipp et al., 2024).

Stiff ECM:

- Increases cell adhesion and spreading.
- Induces changes in cell morphology (flattened, more spread out).
- However, if stiffness is excessive, pore size decreases, physically blocking cell migration (Tien et al., 2020; Saif Ur Rahman et al., 2023).

Intermediate stiffness:

- Provides enough mechanical cues to promote detachment and migration.
- Optimal pore size allows cells to adopt amoeboid movement and efficiently escape the TME (Geiger et al., 2019; Tien et al., 2020).

Fibrosis within the TME is a major driver of cancer progression, immune evasion, and resistance to therapy (Chen et al., 2024). By creating physical, metabolic, and signaling barriers, fibrosis orchestrates a microenvironment that shields tumor cells from immune attack and diminishes the efficacy of conventional and novel cancer therapies (Chen et al., 2024; Tajaldini et al., 2023; Jiang et al., 2017). Understanding and targeting the fibrotic components of the TME are crucial for the development of more effective cancer treatments. One of the most significant consequences of tumor fibrosis is the development of hypoxia within the TME. When fibrosis becomes extensive, tumor tissues often become poorly innervated with blood vessels, creating a highly hypoxic environment with limited access to nutrients and altered cellular metabolism (Huang et al., 2020). This fibrosis-induced hypoxia serves as an important modulator of tumor immunity, creating conditions that favor tumor progression (Jiang et al., 2017). Evidence indicates a positive feedback loop between fibrosis and hypoxia in certain tumor types. Hypoxia can induce deposition of ECM in hypoxic tumor regions, while fibrosis creates further hypoxic conditions (Huang et al., 2020). Recent studies have uncovered mechanisms wherein hypoxia induces fibrosis by increasing the expression of collagen genes and intracellular/extracellular collagen-modifying enzymes, thus perpetuating this cycle (Huang et al., 2020).

In summary, cancer progression and treatment response are significantly influenced by the TME, with fibrosis emerging as a critical factor in immune evasion and therapy resistance. In tumors, excessive collagen deposition leads to a denser and stiffer ECM, a hallmark of many solid cancers such as breast and pancreatic tumors. This increased stiffness influences cancer cell behavior, promoting migration, invasion, and metastasis.

#### 5.6.9 Tumors and fibrosis: a complex relationship

Fibrotic diseases, characterized by excessive accumulation of ECM components, are one of the leading causes of mortality, accounting for 35.4% of global deaths (Mutsaers et al., 2023). Next to conditions such as pulmonary fibrosis and cirrhosis, cancer growth and metastasis driven by fibrotic mechanisms contribute significantly to the aforementioned mortality rates (Mutsaers et al., 2023).

### 5.7 Tumor progression and microenvironmental consequences

Collagen remodeling not only provides a structural scaffold for tumor cell movement and metastasis but also stores and regulates the availability of growth factors and cytokines in the ECM, impacting tumor proliferation and immunomodulation (Boyd and Thomas, 2017). The interaction of collagen, fibroblasts, and immune cells constitutes a feedback loop: tumor cells mechanically and enzymatically alter ECM collagen, which, in turn, modifies cell migration, immune landscape, and treatment response (Lo Buglio et al., 2024; Du et al., 2023; Perentes et al., 2008). The orientation of collagen also affects cellular adhesion signals, influencing integrin-mediated migration and chemokine gradient navigation for both tumor and immune cells (Du et al., 2023; Miyazaki et al., 2019). Such collagen-rich, reorganized microenvironments are prognostic in several cancers and are under investigation as therapeutic targets and diagnostic biomarkers (Lo Buglio et al., 2024; Zhou et al., 2025).

Collagenous tissue is not just a passive scaffold but an active participant in tumor progression. Its tumor-induced remodeling, alignment, and altered porosity regulate both tumor cell invasion and immune cell trafficking, acting as both a guidewire and barrier within the ECM (Fang et al., 2014; Li et al., 2017; Du et al., 2023; Perentes et al., 2008; Morkunas et al., 2021).

#### 5.7.1 How tumor cells use tissue clefts to move

Tumor cells exploit tissue clefts—pre-existing or remodeled microchannels and gaps within the tissue’s ECM—to move and spread within and beyond their primary site. The clefts, comparable to a city’s network of roads leading to a central highway, serve as physical conduits that facilitate cell migration.

Tumor cells often migrate along these pre-formed or remodeled clefts because they provide paths of lower mechanical resistance compared to densely packed surrounding matrix. This enables faster, less energy-intensive movement, sometimes even in a way that bypasses the need for proteolytic (enzyme-driven) ECM degradation (Carey et al., 2015). The size and shape of clefts influence whether a tumor cell must deform its nucleus and cytoskeleton to squeeze through, or whether it can pass more freely. These adaptations are pivotal for successful invasion and subsequent metastasis (Krause and Wolf, 2015). In muscle and other tissues, clefts and aligned ECM fibers can guide tumor cells, similar to how roads channel traffic, increasing invasiveness and metastatic spread, especially when these conduits connect to lymphatics or blood vessels (Beunk et al., 2019; Carey et al., 2015). Some tumor cells can alternate between proteolytic and non-proteolytic migration, taking advantage of clefts when available or creating new ones by degrading the ECM when necessary (Bradbury et al., 2012; Carey et al., 2015).

#### 5.7.2 Physiological role of tissue clefts

In healthy tissue, these clefts serve important homeostatic functions. They act as channels that allow rapid movement of interstitial fluid, contributing to tissue hydration and nutrient distribution. Immune cells use these same clefts as conduits to navigate through tissues, reach sites of infection or injury, and perform immune surveillance. Their movement, like tumor cells, often follows the path of least resistance—squeezing through intercellular junctions and tissue spaces. Thus, clefts balance tissue function by enabling both fluid movement and immune cell mobility.

#### 5.7.3 Cancer progression—a double-edged sword

The existence of these clefts, crucial for physiological function, unfortunately also provides cancer cells with ready-made highways to escape the primary tumor and invade remote tissues. This dual-use nature underlines why metastatic cancer is so challenging to treat: blocking these conduits to hinder cancer spread risks interfering with essential tissue processes like immune defense and fluid regulation (Carey et al., 2015; Winkler et al., 2020).

In summary, tissue clefts act as both a facilitator of homeostasis and an enabler of tumor progression—forming the microscopic “roadways” that both maintain healthy tissue and provide metastatic tumor cells with a route to spread.

## 6 The cancer connection: from normal function to pathological role

In healthy tissues, fibroblasts are quiescent cells found in the stroma. When tissue injury occurs, they activate, proliferate, and contribute to wound healing by producing ECM proteins such as collagen, laminins, and fibronectin. Activated fibroblasts, sometimes called myofibroblasts, also express alpha smooth muscle actin (α-SMA) and help contract and remodel the ECM, supporting tissue repair. After repair, their numbers diminish via programmed cell death (nemosis), restoring tissue homeostasis (Toledo et al., 2022; Yang et al., 2023).

In early tumorigenesis, normal fibroblasts can have a protective role against malignant transformation, inducing cancer cell apoptosis through reactive oxygen species (ROS)-mediated pathways. They regulate ECM turnover and help maintain the architecture and anti-tumorigenic microenvironment (Chhabra and Weeraratna, 2023; Toledo et al., 2022).

During cancer progression, the wound-healing traits of fibroblasts are hijacked. Persistent signals from tumor cells (like TGF-β, IL-6, PDGF, growth factors, and exosomal miRNAs) chronically activate fibroblasts, converting them into CAFs. CAFs avoid apoptosis and become a functionally diverse, proliferative population that supports tumor growth and invasion (Asif et al., 2021; Yang et al., 2023; Sahai et al., 2020; Toledo et al., 2022).

CAFs produce excessive ECM components, matrix metalloproteinases (MMPs), and signaling molecules that remodel the TME and facilitate cancer cell migration, angiogenesis, and EMT. Chronic inflammation, common in tumors, is further driven by cytokines (IL-6, TGF-β, NF-κB), enhancing genomic instability and immune evasion and promoting stem cell-like traits in tumor cells (Lee et al., 2024; Sahai et al., 2020; Toledo et al., 2022).

While fibroblasts start as defenders against malignancy, their gradual conversion to CAFs shifts their function to tumor promotion. Signals such as SLIT2 initially suppress tumor growth, but as cancer progresses, CAFs decrease SLIT2 and increase CXCL12, which drives metastasis. Tumor cells also secrete factors that perpetuate CAF activation and further malignant progression (Toledo et al., 2022).

### 6.1 Clinical relevance

Non-healing wound analogy: Tumors have been likened to “non-healing wounds” where normal mechanisms of tissue repair are subverted, leading to persistent fibroblast activation and chronic, abnormal tissue remodeling. This analogy highlights the similarities between wound healing and stroma-driven cancer progression (Yang et al., 2023; Toledo et al., 2022).

Therapeutic targeting: Because CAFs can promote chemo- and immunotherapy resistance, targeting their signaling pathways (e.g., TGF-β, IL-6/STAT3, ROS, and ECM remodeling enzymes) is an area of active research for new cancer therapies and improving patient outcomes (Chuangchot et al., 2023; Sahai et al., 2020; Toledo et al., 2022).

Fibroblasts maintain tissue health and suppress cancer early on, but become co-opted during cancer progression into CAFs, aiding tumor growth, invasion, and immune evasion via sustained molecular and cellular interactions within the TME (Sahai et al., 2020; Chhabra and Weeraratna, 2023; Yang et al., 2023; Toledo et al., 2022).

Fibroblast-to-myofibroblast transition (FMT) is a key cellular process in wound healing and fibrosis where fibroblasts, which are responsible for producing and maintaining the extracellular matrix (ECM), become myofibroblasts. This transition involves both chemical and physical cues and is central to tissue regeneration and pathological fibrotic responses. FMT is governed by a complex interplay of biochemical and mechanical cues. Understanding and manipulating these signals—which range from growth factor signaling to the mechanical properties of the cellular microenvironment—are critical in both tissue engineering (regeneration) and treatment of fibrotic diseases (D'Urso and Kurniawan, 2020).

**Figure 4:**
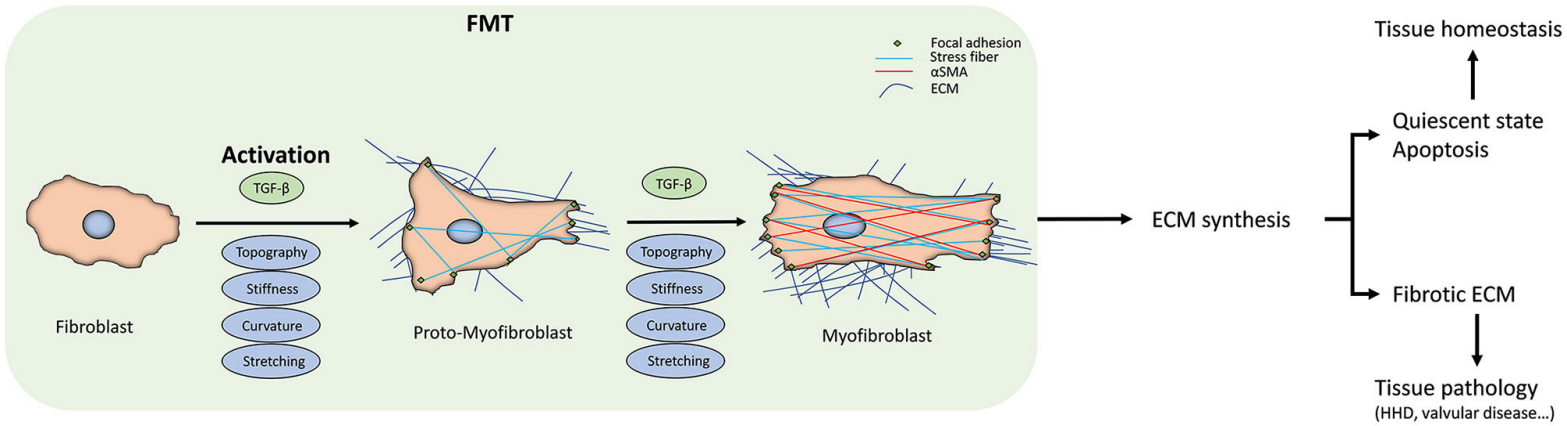
Fibroblast-to-myofibroblast transition (FMT) (D'Urso and Kurniawan, 2020). Copyright ^©^2020, D’Urso and Kurniawan. This is an open-access article distributed under the terms of the Creative Commons Attribution License (CC BY). The scheme summarizes the FMT process, the corresponding changes in fibroblast behavior, and the downstream effects at the tissue level. The transition starts from fibroblast activation due to different kinds of stimuli. The activation can sometimes be reversed or can proceed to the apoptosis of the myofibroblasts. When they escape these routes, due either to the persistent stimuli or to intracellular misregulations, FMT will lead to changes in the extracellular matrix (ECM) deposition and its architecture, driving the tissue to a pathological state. At the cellular level, FMT yields noticeable effects on intracellular stress fibers and α-SMA expression (Beaven et al., 2022).

The scheme summarizes the FMT process (**Figure 4**), the corresponding changes in fibroblast behavior, and the downstream effects at the tissue level. The transition starts from the fibroblast activation due to the different kinds of stimuli. The activation can sometimes be reversed or can proceed to the apoptosis of the myofibroblasts. When they escape these routes, due either to the persistent stimuli or to intracellular misregulations, FMT will lead to changes in the extracellular matrix (ECM) deposition and its architecture, driving the tissue to a pathological state. At the cellular level, FMT results in appreciable in the intracellular stress fibers and α-SMA expression.

S100A4: The transition from normal physiological function to cancer-promoting activity represents one of the most intriguing aspects of fasciacytes biology. The key molecular link lies in the S100A4 protein, which fasciacytes express as part of their normal phenotype but which becomes a powerful driver of cancer progression when dysregulated (Fei et al., 2017). S100A4, also known as metastasin (Mts1), has emerged as a central player in cancer metastasis across multiple tumor types. Extensive research has demonstrated that S100A4 overexpression is strongly associated with poor clinical outcomes in patients with pancreatic, breast, lung, bladder, and gastric cancers (Fei et al., 2017).

The protein functions through both intracellular and extracellular mechanisms to promote cancer progression. Intracellularly, S100A4 enhances the stability of lamellipodia and promotes chemotactic cell migration through interactions with non-muscle myosin IIA (Fei et al., 2017). This activity directly contributes to increased tumor cell motility and invasiveness. The protein also activates critical signaling pathways including Src and focal adhesion kinase (FAK), which are essential for cancer cell survival, proliferation, and metastasis (Tan et al., 2023).

Extracellularly, S100A4 released by fasciacytes and other cells alters the TME in ways that promote cancer progression. It stimulates angiogenesis, attracts immune cells to tumor sites, and promotes the secretion of various cytokines and growth factors (Stecco et al., 2018). The protein can also interact with the receptor for advanced glycation end products (RAGE), triggering downstream signaling cascades that support cancer cell survival and proliferation (Stecco et al., 2018).

Hyaluronan and the TME: The hyaluronan produced by fasciacytes plays a complex and dual role in cancer progression. This complexity stems from the fact that hyaluronan’s biological effects are highly dependent on its molecular weight and the specific context in which it is encountered (Slater et al., 2024).

High molecular weight hyaluronan (HMW-HA, >1,000 kDa) generally exhibits anti-oncogenic properties. It activates the tumor-suppressive Hippo pathway through CD44 receptor engagement, leading to growth inhibition and apoptosis in cancer cells. This anti-cancer effect is exemplified by naked mole rats, which produce unusually large HA polymers and show remarkable resistance to cancer development. In contrast, low molecular weight hyaluronan (LMW-HA, <10 kDa) promotes cancer progression through multiple mechanisms (Donelan et al., 2022). LMW-HA fragments, produced through hyaluronidase-mediated degradation, compete with HMW-HA for CD44 binding and inhibit the protective Hippo signaling pathway. These fragments also stimulate cancer-related inflammation, promote angiogenesis, and facilitate metastatic cell spreading (Donelan et al., 2022).

The TME is characterized by aberrant hyaluronan metabolism, with many solid cancers showing increased HA production accompanied by enhanced degradation due to elevated hyaluronidase activity. This altered metabolism creates an environment rich in pro-inflammatory HA fragments that drive cancer progression while simultaneously depleting the protective HMW-HA (Donelan et al., 2022).

### 6.2 Fascia in special conditions during and after cancer treatment

#### 6.2.1 Fasciacytes in chemotherapy-induced complications

Recent research has revealed an unexpected link between fasciacytes and chemotherapy-induced peripheral neuropathy (CIPN), a common and debilitating side effect of cancer treatment (Wang et al., 2023). Rats were intraperitoneally administered with vincristine (VCR) (Wang et al., 2023), a commonly used anticancer drug leading to musculoskeletal disorders with symptoms like sensory pain, altered touch sensation, numbness, and tingling (Taillibert et al., 2016). Moreover, the researchers observed a decrease in hyaluronan synthase (HAS) expression and a loss of HA-rich fasciacytes in the fascia (Wang et al., 2023).

While CIPN has traditionally been attributed to direct nerve damage, these findings highlight fascia and fasciacytes as potential non-neuronal contributors to the condition. Therefore, fascia could be the cause of musculoskeletal pain in patients with CIPN due to fascial sliding defect and impaired entire function of the tissue (Wang et al., 2023). Damaged fascia and reduced hyaluronic acid may lead to impaired tissue gliding, increased stiffness, and pain, adding a new dimension to the understanding and potential treatment of CIPN (Wang et al., 2023). So far, there are hardly any recommendations regarding CIPN. This opens new avenues for therapeutic interventions targeting the fascia and its cellular components, such as treatments aimed at restoring hyaluronic acid levels or protecting fasciacytes during chemotherapy. The fasciacyte dysfunction appears to contribute to the musculoskeletal pain and mechanical hypersensitivity observed in CIPN patients. The reduction in hyaluronan production impairs fascial gliding and alters tissue viscoelasticity, potentially activating nociceptors and contributing to the pain symptoms traditionally attributed solely to neuronal damage. This finding suggests that the symptoms of CIPN may be partially due to fascial dysfunction rather than purely neurological causes.

The discovery of fasciacytes has fundamentally transformed our understanding of both fascial biology and cancer progression. These specialized cells represent a unique example of how normal physiological functions can be co-opted by cancer cells to promote disease progression. The S100A4 protein and hyaluronan production by fasciacytes create a complex web of interactions that influence cancer cell behavior, TME dynamics, and treatment responses.

The therapeutic potential of targeting fasciacytes is substantial, with multiple approaches showing promise in preclinical and early clinical studies. However, the challenge lies in developing treatments that can effectively target the cancer-promoting functions of fasciacytes while preserving their essential roles in normal tissue function. The emerging understanding of fasciacyte mechanobiology and their role in chemotherapy-induced complications adds additional layers of complexity and opportunity to this field.

As we move forward, the integration of fasciacyte research with broader cancer biology and treatment development promises to yield new insights and therapeutic approaches. The field stands at an exciting juncture where fundamental discoveries about fascial biology are translating into practical advances in cancer diagnosis, prognosis, and treatment. The continued investigation of fasciacytes will likely yield significant benefits for cancer patients while advancing our understanding of the intricate relationships between connective tissue biology and human disease.

#### 6.2.2 Graft versus host disease

Graft-versus-host disease (GVHD) is a complex immune-mediated complication after allogeneic stem cell transplantation, with acute and chronic phases affecting multiple organs. Its management requires careful clinical assessment and immunosuppressive therapy, with ongoing research to optimize the beneficial effect while minimizing harm from GVHD.

Chronic GVHD is believed to arise when the donor’s immune system identifies recipient tissues, leading to inflammation and fibrosis. While joint/fascia manifestations in chronic GVHD patients are deemed uncommon, there has been a lack of research into this complication. Although the muscle-related complications of fasciitis and myositis resulting from chronic GVHD after Allo-SCT are not common, they can significantly affect a patient’s quality of life (QOL) (Oda et al., 2009; Shimizu et al., 2021). Joint and fascia involvement occurs in approximately 3%–29% of patients with chronic GVHD (Inamoto et al., 2014). Furthermore, 10%–15% of GVHD patients develop sclerotic features affecting the skin or deeper tissues, leading to functional limitations and poor quality of life (Molés-Poveda et al., 2021). Joint/fascia manifestations that have been reported consist of joint stiffness, edema, restricted range of motion (ROM), arthralgia, and, on rare occasions, arthritis or synovitis (Inamoto et al., 2014).

Treatment:

- Corticosteroids are typically the first-line treatment, but their effectiveness can be limited, especially in steroid-resistant cases (Shimizu et al., 2021).
- Other therapies, such as JAK inhibitors (e.g., baricitinib), have shown promise in steroid-resistant cases (Shimizu et al., 2021).
- Physical therapy, stretching, and occupational therapy are important for maintaining mobility and function.
- Assistive devices, splints, and deep tissue massage may help relieve symptoms and improve range of motion.

In summary, fascia involvement in chronic GVHD is a recognized and potentially debilitating complication characterized by inflammation, fibrosis, and restricted movement, primarily affecting the joints and soft tissues. Early recognition and multidisciplinary management are crucial to minimize disability and improve patient outcomes (Inamoto et al., 2014; Shimizu et al., 2021).

For leukemia patients, myofascial release therapy is beneficial, especially in managing treatment-related side effects, such as those due to chemotherapy and radiation. These therapies may cause muscle and fascia tightness, discomfort, and restricted mobility. Physical therapy focusing on fascia, like myofascial release, can help reduce pain, improve mobility, alleviate fatigue, and enhance the sleep quality for leukemia patients. Damage to the fascia due to medical treatments, including fibrosis, may reduce mobility and worsen posture, but targeted therapy can help restore fascia length and flexibility (Weigmann-Faßbender et al., 2022).

## 7 What role does tissue stiffness play in tumor progression and resistance?

Tissue stiffness is a central mechanical feature of the TME that profoundly influences tumor progression and resistance to therapy. Increased stiffness is driven by excessive ECM deposition, contraction, and cross-linking, primarily orchestrated by cancer-associated fibroblasts (CAFs) and tumor cells themselves (Ishihara and Haga, 2022; Huang et al., 2021; Yui and Oudin, 2024).

This stiffened environment promotes several key aspects of tumor biology, namely:

- Tumor proliferation and invasion: Stiff matrices activate signaling pathways such as integrin-mediated focal adhesion kinase (FAK), PI3K/Akt, and Ras–MAPK, which drive tumor cell proliferation, migration, and invasion (Huang et al., 2021; Yui and Oudin, 2024; Liang and Song, 2023). The Hippo pathway, particularly YAP/TAZ transcription factors, is also activated by stiffness, promoting the expression of genes involved in cell proliferation and survival (Huang et al., 2021).
- EMT: Stiffness induces EMT, a process where tumor cells lose cell-to-cell adhesion and gain invasive properties, facilitating metastasis (Deville and Cordes, 2019; Liang and Song, 2023; Wei and Yang, 2016). This is mediated by mechanotransduction signals and changes in microRNA and protein expression.
- Resistance to therapy: Stiff TME contributes to resistance against chemotherapy, radiation, and targeted therapies. Mechanistically, stiff ECM enhances cell-adhesion-mediated drug resistance (CAM-DR) and radioresistance (CAM-RR) through integrin and focal adhesion signaling (Deville and Cordes, 2019; Huang et al., 2021). Additionally, stiff matrices impede immune cell infiltration, reducing the effectiveness of immunotherapy (Mai et al., 2024; Yui and Oudin, 2024).
- Angiogenesis and hypoxia: Increased stiffness can promote angiogenesis (formation of new blood vessels) but also leads to high interstitial pressure and hypoxia, both of which are associated with therapy resistance and poor prognosis (Deville and Cordes, 2019; Yui and Oudin, 2024).
- Genomic instability and aggressiveness: Stiff environments can cause nuclear envelope rupture and DNA damage during cancer cell migration, contributing to genomic instability and the emergence of more aggressive tumor clones (Yui and Oudin, 2024; Deville and Cordes, 2019). Tumors in stiff tissues tend to accumulate more mutations and display greater genomic diversity.

**Table 6** gives an overview of the effects of tissue stiffness on tumors.

**Table 6:** Evidence levels for key fascia-cancer mechanisms.

Mechanism/claim	*In vitro*	Animal	Human clinical	Evidence level
ECM stiffness → integrin clustering → FAK/ROCK activation	Strong (Paszek and Weaver, 2004; Baker and Zaman, 2010)	Moderate	Indirect (IHC correlations)	Preclinical—strong
ECM stiffness → YAP/TAZ nuclear translocation → EMT	Strong (Dupont et al., 2011; Di et al., 2023)	Moderate (conditional KO models)	Indirect correlative data	Preclinical—strong
Matrix stiffness → TWIST1 release from G3BP2 → EMT	Strong (Wei et al., 2015; Fattet et al., 2020)	Moderate	Collagen fiber alignment predicts survival in breast CA	Translational—moderate
CAF-mediated LOX/LOXL2 cross-linking increases stiffness	Strong (Kirschmann et al., 2002; Erler and Weaver, 2009)	Strong	Clinical correlations in breast, PDAC (Quintela-Fandino et al., 2024)	Translational—strong
Fascial infiltration as independent prognostic marker	N/A	N/A	Sarcoma cohorts (Pasquali et al., 2018; Lee et al., 2020)	Clinical—moderate
Fasciacyte S100A4 promotes cancer cell migration	Moderate (Wang et al., 2023)	Limited	None	Preclinical—limited
Fascial stiffening drives tumor invasion (causal)	Strong (tunable hydrogels)	Moderate (Berrueta et al., 2018a; Langevin et al., 2016, 2016)	None	Preclinical—moderate
Stretching reduces tumor growth	None	Yes (Berrueta et al., 2018a; He et al., 2025)	None (QoL data only)	Preclinical—hypothesis-generating
Solid stress reduction improves drug delivery	Strong	Strong (Papageorgis et al., 2017; Mpekris et al., 2018)	Phase II sarcoma (Panagi et al., 2024)	Translational—emerging
LOX inhibition improves therapy response	Moderate	Strong (BAPN, LOXL2 inh.; (Yui and Oudin, 2024)	Phase I/II ongoing	Translational—emerging
Fascial therapies improve drug delivery in humans	None	None	No RCT data	Hypothesis—unvalidated
TGF-β inhibition + checkpoint blockade	Strong	Strong	Phase I/II early data (Metropulos et al., 2022)	Translational—emerging

In summary, tissue stiffness is a critical driver of tumor progression and resistance by promoting proliferation, invasion, therapy resistance, immune evasion, and genomic instability (Liang and Song, 2023; Deville and Cordes, 2019; Huang et al., 2021). Targeting ECM stiffness is an emerging therapeutic strategy to improve cancer outcomes. Research suggests that therapies targeting fascia—such as mechanotherapy, antifibrotic drugs, and CAF-directed immunotherapy—could influence cancer progression and metastasis.

## 8 Open research questions on connective tissue in cancer biology

Connective tissue, long considered a passive structural component, is now recognized as a dynamic player in cancer biology. Its roles extend far beyond mere support, influencing tumor growth, progression, metastasis, and even patient prognosis. The “mystics” of connective tissue in cancer refer to the complex, sometimes paradoxical, and still not fully understood ways in which these tissues interact with cancer cells and the TME. Connective tissue is a central, enigmatic force in cancer biology, shaping the fate of tumors through its cellular diversity, signaling complexity, and physical properties. Its dual nature—as both a shield and a conduit for cancer—makes it a compelling focus for future research and therapy (Langevin et al., 2016; Bartoschek et al., 2018).

Fascia is no longer seen as a mere anatomical bystander but as a dynamic, influential player in cancer biology. Its state of health—particularly in terms of inflammation and stiffness—may affect cancer risk, progression, and recovery. Integrative therapies targeting the fascia offer promising adjuncts to conventional cancer care, but further research is needed to fully understand their benefits and risks (Langevin et al., 2016).

Dense breasts and inflammation are linked to poorer breast cancer prognosis through complex interactions between collagen-rich ECM components and immune cells. High mammographic density, characterized by increased stromal collagen, creates a pro-TME where macrophages interact with ECM collagen to promote chronic inflammation and tumor progression. This inflammatory response involves elevated COX-2 levels and altered collagen structures that correlate with tumor recurrence and reduced survival. Preclinical studies suggest that COX-2 inhibitors like celecoxib may improve the outcomes in dense-breast patients by reducing inflammation and ECM remodeling. The connective tissue growth factor (CTGF) plays a parallel role in pancreatic cancer progression (Bennewith et al., 2009). CTGF overexpression enhances anchorage-independent tumor growth and metastasis, while CTGF-specific monoclonal antibodies (e.g., FG-3019) inhibit these processes by blocking CTGF-mediated signaling pathways (Jacobson and Cunningham, 2012). This demonstrates the therapeutic potential of targeting ECM-associated growth factors.

Hyaluronan is essential for tissue health and function. Its dysregulation can contribute to pain, swelling, and disease processes, including cancer. Dysregulated hyaluronan metabolism is associated with cancer development. Excessive hyaluronan can lead to tissue swelling, increased interstitial pressure, and compression of neurovascular structures, resulting in pain and impaired function (Slater et al., 2024). Hyaluronan (HA) further exemplifies ECM–tumor interactions.

Key findings

Pro-angiogenic effects: Low molecular weight HA fragments promote lymphangiogenesis via LYVE-1 receptor signaling, facilitating metastatic spread. Hyal-1 over-expression has been reported to promote mammary tumor growth and increased tumor angiogenesis in animal models. In this context, increased hyaluronan levels may also serve as a diagnostic marker for some cancers (Monslow et al., 2015).

Immune modulation: HA degradation produces a pro-inflammatory milieu that recruits immunosuppressive myeloid-derived suppressor cells (MDSCs) and promotes PD-L1+ macrophage polarization. It has also been shown that HMW-HA was associated with protective effects in tumorigenesis (Mueller et al., 2010).

Clinical impact: HA accumulation correlates with enhanced tumor stroma formation, angiogenesis, and reduced survival across multiple cancers—differentiated/nuanced perspective regarding MW of HA: e.g., relevance of molecular weight in cancer progression; increased HA fragmentation: e.g., reported with prostate, larynx, brain tumor cyst fluid, and colon cancer; no reported change in MW distribution in lung or liver cancer. Varied results were reported in relation to oligo-HA (Monslow et al., 2015).

These mechanisms collectively highlight how ECM components like collagen, CTGF, and HA interact with immune cells to drive tumor progression through inflammation, stromal remodeling, and metastatic signaling. Therapeutic strategies targeting these interactions—such as COX-2 inhibition, CTGF-neutralizing antibodies, or HA metabolism modulations—show promise for improving cancer outcomes.

## 9 Could TME treatment by physical force be an option to mitigate fibrotic diseases or even cancer?

Physical forces can impact cellular behavior and tissue remodeling. The influence of mechanical interventions (e.g., massage, acupuncture) on tumor biology remains an active area of investigation, with potential implications for both therapeutic benefit and unintended risk. Mechanical stimulation serves as a potent modulator of anabolic and catabolic cellular metabolism as well as the physical properties of the extracellular matrix synthesized by cells, ultimately shaping emergent tissue architecture. Mounting evidence positions enhanced stiffness within the connective tissue microenvironment as a pivotal mechanobiological regulator of tumorigenesis and metastatic dissemination. Therapeutic strategies targeting tissue stiffness alongside associated inflammatory signaling may therefore exert beneficial effects on tumor progression by modulating both biomechanical forces and physiological adaptive responses (Langevin et al., 2016). Physical-based therapies manage symptoms and improve side effects from cancer treatment as well as reduce connective tissue inflammation and fibrosis and thus may have direct beneficial effects on cancer spreading and metastasis (Langevin et al., 2016). Standardizing models of mechanotransduction could enhance translational research, leading to more effective treatments for metastatic cancers (Mierke, 2024). Mechanobiological strategies are emerging as innovative approaches to combat cancer by targeting the physical and mechanical properties of tumors and their microenvironment (Kumari et al., 2023). These methods focus on altering the ECM, disrupting mechanotransduction pathways, and leveraging mechanical forces to enhance treatment efficacy (Kumari et al., 2023). Understanding the interplay between ECM mechanics and cancer cell behavior can improve therapeutic strategies. Targeting ECM remodeling or mechanotransduction pathways may help disrupt tumor progression (Mierke, 2024).

## 10 Potential risks and concerns

### 10.1 Safety of mechanical interventions in oncology: a risk-stratified framework

Safety considerations for fascial and manual therapies in cancer patients require explicit, risk-stratified guidance rather than general recommendations. The risk profile varies substantially based on disease stage, treatment phase, tumor location, and the specific intervention applied.

Low-risk settings: In patients with locally resected tumors and no evidence of residual or recurrent disease, gentle movement therapies, including supervised stretching, yoga, and therapeutic massage applied away from the surgical site, are generally considered safe within structured integrative oncology programs. No published study has demonstrated an increase in recurrence rates from these interventions in this population, and QoL benefits are consistently demonstrated (Wang and Zhou, 2021; Kim et al., 2023b). This favorable risk profile supports their integration as standard supportive care.

Moderate-risk settings—active local disease: For patients with active local disease or incomplete surgical resection, direct mechanical manipulation overlying or immediately adjacent to the tumor site raises theoretical concern. It has been postulated that mechanical pressure or shear forces could dislodge malignant cells, potentially encouraging their migration (Langevin et al., 2016; Zhu et al., 2023). While direct human evidence for this specific risk remains absent, the preclinical observations of Langevin et al. (2016) and the general mechanobiological data on force-induced tumor cell displacement (Langevin et al., 2016; Jain et al., 2014) warrant clinical caution. In these settings, oncological multidisciplinary team (MDT) approval should be obtained before initiating manual therapies near active tumor sites, and interventions should be limited to gentle, non-compressive modalities.

High-risk settings, metastatic and treatment-compromised patients: In patients with bone metastases, direct joint manipulation or strong mechanical forces carry the risk of pathological fracture and are contraindicated without imaging confirmation of structural integrity. In patients with thrombocytopenia (platelet count <50 × 10^9^/L) from myelosuppressive treatment, deep massage poses clinically meaningful bleeding risk. In patients with established or at-risk lymphedema, massage must be delivered exclusively by certified lymphedema therapists familiar with oncological contraindications; inappropriate manual lymphatic drainage near tumor-bearing nodal stations may theoretically accelerate lymphatic spread (Kim et al., 2023b). For patients receiving anticoagulant therapy, pressure-based fascial techniques require dose-adjusted risk protocols defined in conjunction with the treating hematologist or oncologist.

### 10.2 General safety principles

We recommend that (1) physical interventions in cancer patients be prescribed within an integrative oncology framework with oncologist approval and MDT oversight, (2) the intensity, site, and timing of mechanical interventions be individually assessed based on disease status and treatment phase, (3) prospective safety monitoring endpoints—including circulating tumor cell counts, imaging-based tumor stability assessment, and adverse event reporting—be incorporated into all future clinical trials evaluating physical fascial therapies in active cancer patients, and (4) the current absence of documented serious harm from gentle interventions not be construed as equivalence with established safety in the absence of adequately powered safety studies. Mechanical interventions near tumors should be considered investigational and conducted within properly monitored clinical trial frameworks wherever possible.

## 11 Conclusion and future research direction

The future of fascia and cancer research lies in uncovering the mechanobiological, immunological, and signaling roles of fascia in the TME, developing targeted therapies, and integrating fascial health into holistic cancer care (Slater et al., 2024; Langevin et al., 2016).

Studies are expected to focus on how fascial remodeling, stiffness, and mechanical properties influence tumor growth, metastasis, and therapy resistance. Understanding the biotensegrity model and how mechanical forces within fascia affect gene expression in cancer cells is a key area (Langevin et al., 2016). The development and application of imaging modalities, such as elastography and MRI diffusion tensor imaging, can enable the visualization of fascial structures and their changes during cancer progression and treatment (Langevin et al., 2016).

Since many CAFs originate from fascial fibroblasts, research will likely focus on therapies that target these cells, such as fibroblast-activated protein inhibitors and CAF-directed immunotherapies, to modulate the TME and improve outcomes (Tao et al., 2017). To better understand cell signaling, investigating how fascia acts as a cell signaler, particularly in the context of inflammation and cancer cell communication, may reveal new therapeutic targets.

Furthermore, the dense population of immune-active cells within fascia might play a role in modulating immune responses to tumors. Future research could explore how fascial inflammation contributes to oncogenesis and how targeting this inflammation might prevent or treat cancer (Slater et al., 2024). Studies may also assess the efficacy of anti-inflammatory and mast cell stabilizing treatments applied to fascia in reducing cancer-promoting inflammation (Slater et al., 2024).

There is growing interest in whether therapies such as yoga, acupuncture, and massage—which target fascial health—can reduce fascial stiffness and inflammation and potentially inhibit tumor progression or improve the quality of life for cancer patients. Rigorous clinical trials are needed to validate these approaches and address concerns about safety and efficacy, also systemically by investigating how fascial therapies influence systemic immune modulation and emotional well-being in cancer patients.

Another potential lies in diagnostic innovation and therapeutic targets. Research may seek to identify fascial biomarkers for early cancer detection, prognosis, or monitoring treatment response. Exploring drugs or interventions that specifically target fascial components (e.g., ECM stiffness, collagen cross-linking) may help disrupt tumor-supportive environments (Langevin et al., 2016).

There is a need for basic and translational research to clarify how changes in fascial structure and function directly contribute to cancer initiation, progression, and metastasis (Langevin et al., 2016). High-quality clinical studies are required to translate findings from animal models and *in vitro* studies to patient care, particularly regarding the safety and effectiveness of fascial interventions in oncology (Langevin et al., 2016). Therefore, further studies are needed to evaluate the safety and potential benefits of physical and manual therapies in cancer treatment and to investigate the direct effects of mechanical forces on tumor growth and recurrence. Lastly, it might be interesting to explore the relationship between whole-body connective tissue stiffness and cancer progression.

Progress will depend on the collaboration among oncologists, fascia researchers, immunologists, biomechanical engineers, and integrative medicine practitioners in developing holistic approaches to cancer treatment. Studies should focus on systems-based approaches to emphasize the interconnectedness of fascia with metabolic, immune, and nervous system regulation in cancer biology (Slater et al., 2024).

As research in fascia biology advances, it may provide new insights into integrative oncology approaches and potentially lead to the development of physical treatments that enhance natural healing responses in cancer patients in addition to modern cancer treatment.
